# Quantum-accurate enhanced sampling and mini-protein folding through replica exchange for multiscale neural network potentials

**DOI:** 10.1039/d6sc04555c

**Published:** 2026-11-09

**Authors:** Riccardo Solazzo, Igor Gordiy, Sereina Riniker

**Affiliations:** a Department of Chemistry and Applied Biosciences, ETH Zürich Vladimir-Prelog-Weg 2 8093 Zürich Switzerland sriniker@ethz.ch

## Abstract

Neural network potentials (NNPs) have emerged as efficient alternatives to computationally demanding quantum-mechanical (QM) methods. AMPv3-EE-BMS25 (short AMP-BMS) is an equivariant NNP designed for multiscale ML/MM simulations that captures directional and long-range interactions. In classical molecular dynamics (MD) simulations, enhanced-sampling techniques are applied to cross large energy barriers and improve convergence. Among those, replica exchange with solute tempering (REST2) is an efficient method that selectively scales solute interactions while keeping the bath temperature fixed, thereby reducing the number of replicas required. Here, we present a REST2-like algorithm compatible with multiscale ML/MM simulations. This algorithm is combined with AMP-BMS by exploiting the separability of its Hamiltonian into solute–solute, solute–solvent, and solvent–solvent contributions. We validate the REST2–AMP approach on several peptidic systems in explicit solvent and demonstrate folding of the mini-proteins chignolin and CLN025 from extended conformations. Thus, our REST2 approach for ML/MM enables access to biologically relevant timescales while maintaining near-DFT accuracy.

## Abstract 2

REST2 enhanced sampling has been adapted to electrostatic-embedding ML/MM simulations, enabling mini-protein folding and protein structure refinement at near-DFT accuracy without predefined reaction coordinates.

## Graphical abstract

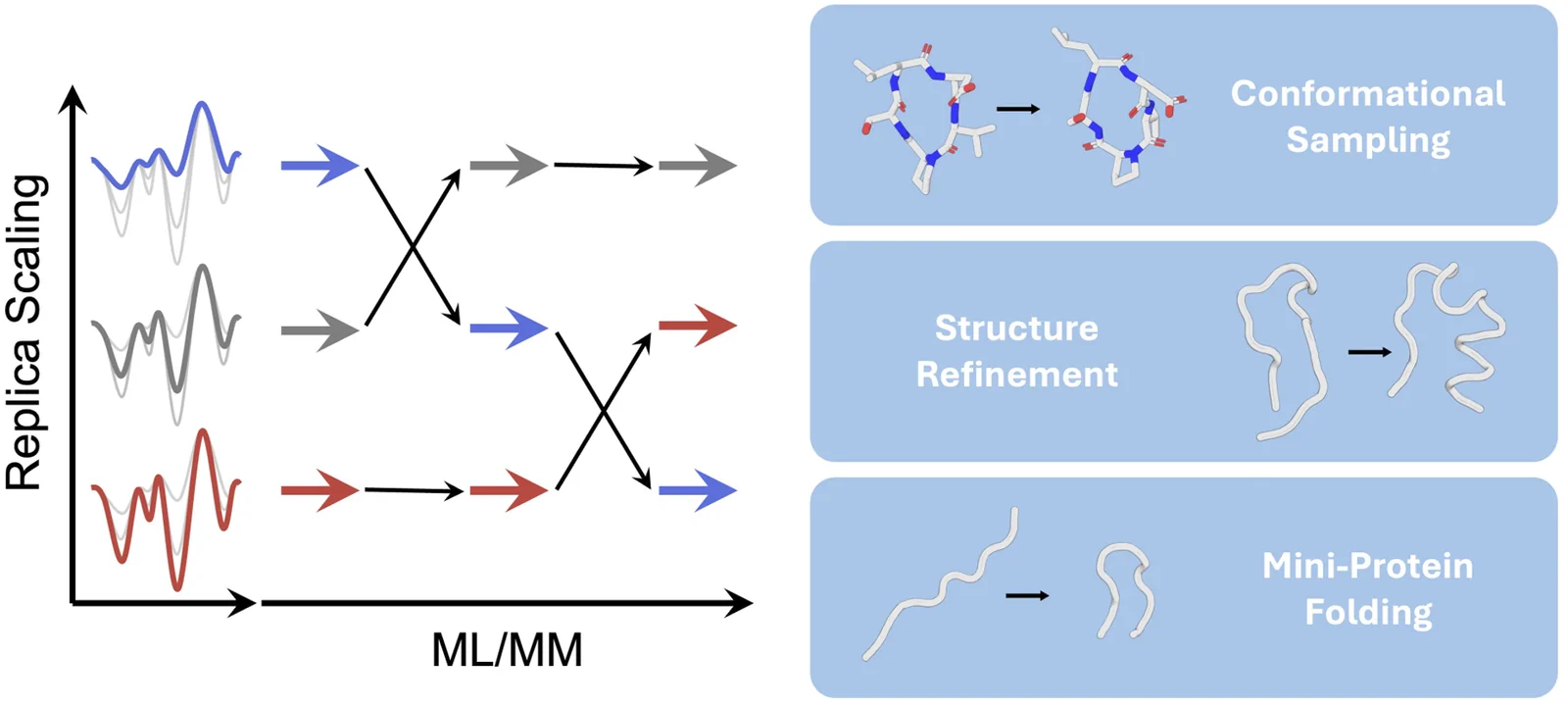

## Introduction

Accurate modeling of biomolecular systems at the quantum-mechanical (QM) level provides critical insights into structural dynamics, reactivity, and noncovalent interactions, which are difficult to capture with classical molecular mechanics (MM) force fields.^1^ However, treating the full simulation box at the same QM level of theory is prohibitively expensive for the majority of systems of interest. In 1976 Levitt, Warshel, and Karplus proposed the multiscale QM/MM scheme that retains QM accuracy in the key region of the system (the QM zone), while describing the surrounding environment (the MM zone, such as solvent molecules and/or distant protein residues in enzymatic reactions) using computationally less expensive classical force fields.^2–7^ Even with the cost reduction thanks to the multiscale scheme, QM/MM simulations remain computationally demanding and scale poorly with the size of the system, limiting the accessible timescales to picoseconds or low nanoseconds depending on the choice of QM method.^8,9^

Over the past decade, computational chemistry has witnessed the emergence of machine learning (ML) models to replace computationally expensive QM Hamiltonians. These models take atomic coordinates, element types, and optionally the molecular charge and spin multiplicity as input and predict the total electronic energy of the system. Such approaches are commonly referred to as neural network potentials (NNPs) or ML interatomic potentials (MLIPs). Since 2011, numerous architectures have been introduced (see ref. 10–23). The integration of NNPs into multiscale frameworks has led to the development of ML/MM approaches, analogous to QM/MM schemes, in which the “QM zone” is described by a NNP while the environment is treated at the MM level.^24–32^ The approaches differ in whether mechanical or electrostatic embedding^4^ is applied to account for the interaction between ML particles and MM particles, whereby the choice of embedding scheme depends on the training data and ML architecture of the model. Among these methods the anisotropic message-passing (AMP) family of models^33–35^ allows for electrostatic embedding and delivers QM-like accuracy while being several orders of magnitude faster than the QM/MM reference based on density functional theory (DFT) that was used for training.

Despite this performance boost, ML/MM molecular dynamics (MD) simulations remain slower than those with classical force fields, which means that the simulation of systems with high (free-)energy barriers like conformational transitions in peptides and proteins will require the use of enhanced-sampling techniques^36–38^ – same as in classical MD.^39,40^ Enhanced-sampling algorithms can be broadly classified into two categories: reaction-coordinate-based methods (*e.g.*, umbrella sampling,^41^ local elevation,^42^ weighted ensemble,^43^ or metadynamics^44^), which enhance sampling along a predefined quantity describing the process of interest, and generalized-ensemble methods, such as temperature replica exchange (TRE)^45,46^ and variants, which enhance sampling across all degrees of freedom of the system. Among the second group, replica exchange with solute tempering (REST2)^47,48^ has found wide usage as it requires fewer replicas than conventional TRE by effectively heating the solute while keeping the solvent at a fixed temperature.

Reaction-coordinate-based approaches like umbrella sampling and metadynamics have been combined with both QM/MM^49–55^ and ML/MM^23,24,28,34,35,56,57^ simulations to investigate chemical reactions and biological processes. However, a key limitation of these methods is the requirement for *a priori* knowledge of the process in order to define suitable reaction coordinates or collective variables.^58–61^ So far, generalized-ensemble methods have been applied to QM/MM simulations, *e.g.*, Yagi *et al.*^62^ implemented generalized replica exchange with solute tempering (gREST) for QM/MM. In addition, Hamiltonian replica exchange has been used in the context of alchemical free-energy calculations with ML/MM.^63^ To the best of our knowledge, no REST(2)-like enhanced sampling scheme has yet been developed for ML/MM simulations.

Here, we propose an approach that combines a REST2-like strategy with the ML/MM framework based on the AMP family of models (Fig. 1A). Compatibility with REST2 requires that the Hamiltonian is separable into solute–solute, solute–solvent, and solvent–solvent contributions, so that the different interactions can be scaled independently.^48^ This separability is exact for classical additive force fields, where the total energy is by construction a sum of pairwise nonbonded and bonded terms unambiguously partitioned by atom membership. The AMP-based ML/MM formulation provides an analogous decomposition into an ML–ML term, describing the intramolecular energy of the ML zone, and an ML–MM term, describing the electrostatic (permanent and induced multipoles) and van der Waals interactions between the ML and MM zones.

**Fig. 1:**
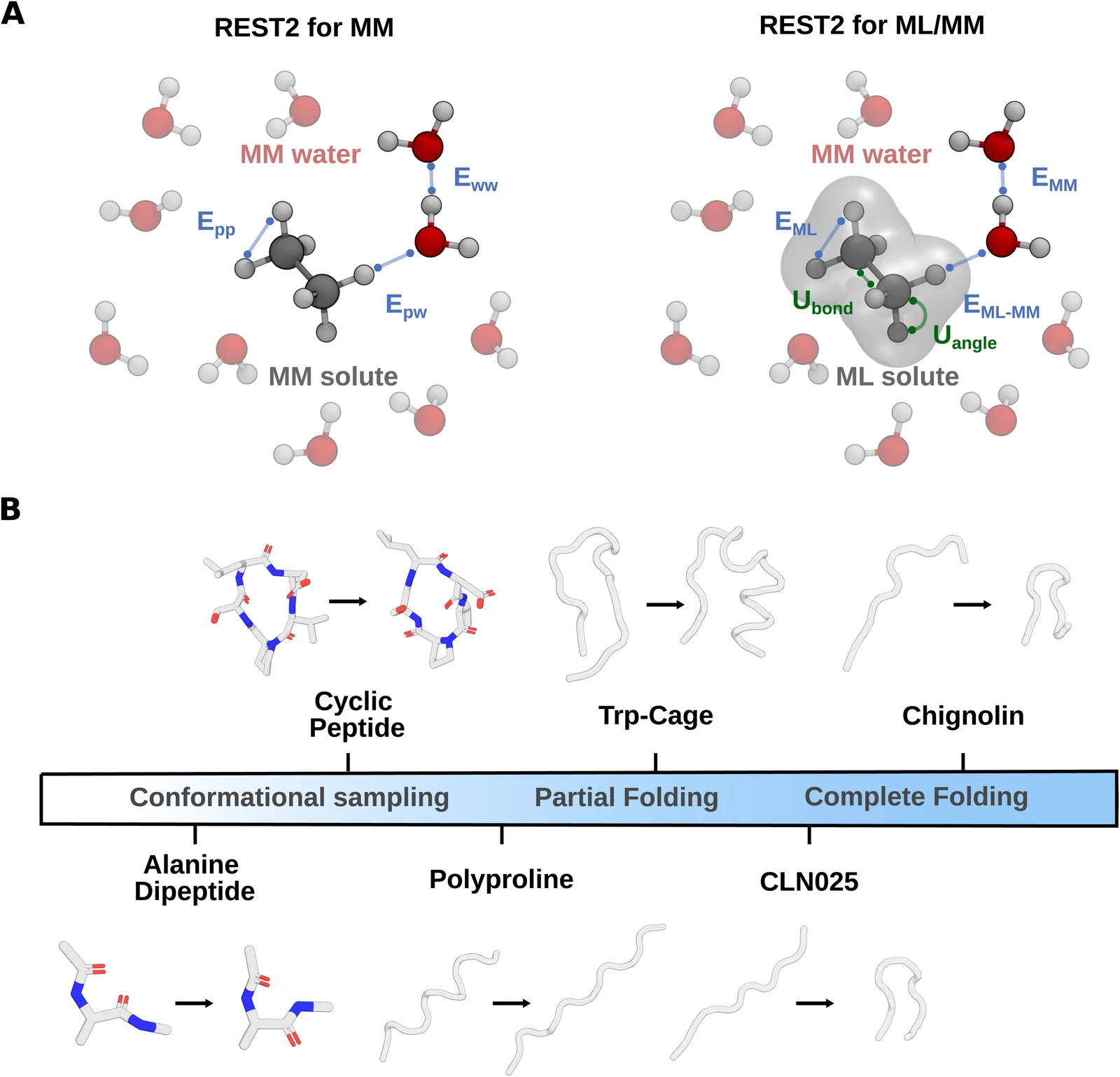
Overview of the REST2 approach for MM and ML/MM frameworks and peptide test systems. (A) Schematic representation of the potential-energy decomposition in classical (MM) simulations *versus* multiscale ML/MM simulations. (B) Studied peptide systems of increasing complexity from alanine dipeptide and cyclic pentapeptide (conformational sampling) to the Trp-cage mini-protein and polyproline (partial folding), and to chignolin and its mutant CLN025 (complete folding).

Unlike in a classical force field, this decomposition in AMP is only approximate: the polarization energy, *i.e.*, the energetic cost associated with the response of the ML zone to the electrostatic environment of the MM region (analogous to electronic polarization in QM/MM) is physically a ML–MM effect, yet it is absorbed into the ML–ML term through the environment-dependent node embeddings, which are updated by the induced multipoles prior to the energy readout (see ref. 35 and 64 for a discussion of polarization in electrostatic-embedding QM/MM and how it relates to ML/MM frameworks). In the AMPv3-EE-BMS25 model^35^ used here, this contribution is controlled by a polarization-scaling parameter *λ*_pol_. At its optimal value (*λ*_pol_ = 0.35), the SI of ref. 35 shows that the magnitude of the polarization energy is negligible relative to *E*_ML–MM_. The ML–ML/ML–MM partitioning is therefore, in practice, an accurate approximation to the solute–solute/solute–solvent split required by REST2.

In contrast, no such decomposition is available for a standard electrostatic-embedding QM/MM Hamiltonian: the intramolecular energy of the QM zone, the polarization energy, and the electrostatic interaction with the MM point charges emerge from a single self-consistent-field (SCF) calculation as one inseparable quantity. Isolating the intramolecular contribution would require either modifying the SCF procedure itself or performing an additional gas-phase calculation of the QM zone, neither of which is available in standard QM/MM implementations. The AMP-based ML/MM Hamiltonian thus occupies an intermediate position between a fully separable classical force field and a non-separable electrostatic-embedding QM/MM Hamiltonian, affording a degree of separability that enables selective solute tempering at an affordable computational cost.

To test the performance of the approach, we make use of the global (or foundational) model AMPv3-EE-BMS25,^35^ which was trained on the BMS25 dataset^65^ for peptides, proteins, and small organic molecules. We will use the short name AMP-BMS in the following. This model can be used for peptidic systems without additional training. The REST2-AMP/MM approach is first applied to two established benchmark systems (Fig. 1B), alanine dipeptide and a cyclic peptide (cyclo-(Pro–Ser–Leu–Asp–Val)), showing that it significantly enhances exploration of the configuration space defined by the dihedral angles. We further demonstrate the ability of REST2-AMP/MM to recover native conformations from partially unfolded or misfolded states. Specifically, we examine a polyproline chain initially modeled as an *α*-helix and a partially unfolded Trp-cage mini-protein in which the *α*-helix was incorrectly modeled as a coil. In both cases, the method successfully refolds the structures toward their native states. Finally, we perform reaction-coordinate-free simulations of chignolin and its mutant CLN025 to evaluate the performance of the method in autonomous folding simulations. Taken together, our results indicate that the approach improves sampling efficiency in small peptides and mini-proteins, allowing high-accuracy structural refinement of such systems. Notably, it enables folding simulations of mini-proteins without requiring prior knowledge of relevant reaction coordinates, while approaching near-DFT accuracy at a fraction of the computational cost required by conventional QM/MM simulations.

## Results and discussion

### Implementation of REST2 in the AMP/MM framework

We implemented a REST2-like replica-exchange scheme within the AMP/MM framework to enable enhanced sampling in a multiscale ML/MM setting. As in REST2,^48^ the central idea is to selectively increase the effective temperature of the solute while maintaining the solvent at the physical temperature.

The AMP/MM Hamiltonian can be decomposed as,

1 *H*(*x*) = *E*_ML_(*x*) + *E*_ML–MM_(*x*) + *E*_MM_(*x*),

where *x* are the coordinates of the system, *E*_ML_ denotes the intramolecular solute energy computed with the NNP, *E*_ML–MM_ the solute–solvent interaction energy described partly by the NNP and partly by the classical force field, and *E*_MM_ the internal solvent energy. In classical REST2, the target scaling of the solute–solute and solute–solvent terms by *γ*_m_ and 
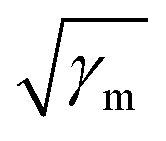
, respectively, is not applied to the energy expression directly. Instead, it is achieved by rescaling the force–field parameters of the solute: partial charges are scaled by 
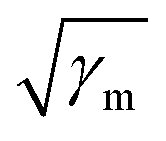
, Lennard-Jones (LJ) *ε*_LJ_ by *γ*_m_, and torsional force constants by *γ*_m_. All solvent parameters are left unchanged. The correct 
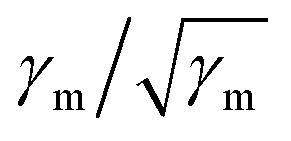
 scaling of the solute–solute and solute–solvent energy terms then follows automatically from the bilinear form of the Coulomb and LJ expressions, the latter *via* the geometric-mean combining rule for *ε*_LJ_.

This parameter-level route is not available to AMP: all multipoles, including monopoles, are predicted internally by the NNP rather than assigned as fixed parameters, and there is no torsional force constant to rescale. We therefore scale the two energy terms directly, after they are computed by the NNP,2
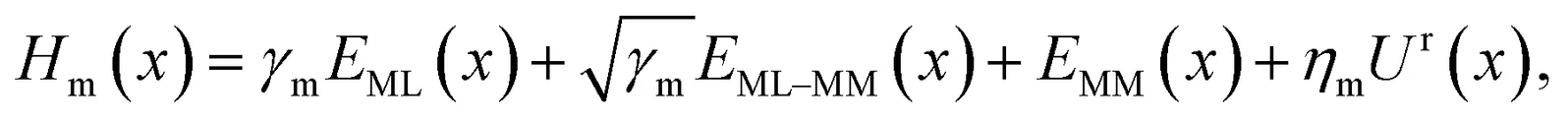
where *γ*_m_ ∈ (0,1] is the replica-dependent scaling parameter and *γ*_1_ = 1 is used for the unscaled reference replica. *E*_ML_(*x*) and *E*_ML–MM_(*x*) are differentiable functions of the coordinates. That is why forces for *H*_m_ are calculated by backpropagation using the scaled Hamiltonian.

*U* ^r^ (with *r* denoting restraints) represents optional scaled harmonic bond and angle restraints, which are applied to prevent unphysical distortions of bonds and angles in highly scaled replicas. *η*_m_ is the second replica-dependent scaling factor that regulates the strength of bond and angle restraints in the scaled replicas (a scheme showcasing the dependence of the scaling factors on a replica index can be found in Fig. S1),3
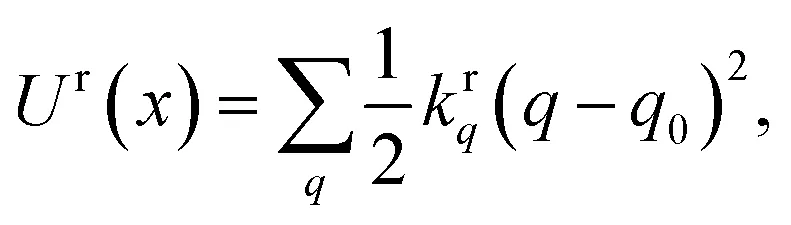
with *q* being a bond length or angle, and *q*_0_ is the corresponding equilibrium value taken from the AMBER ff14SB force field.^66^ The restraint force constant *k*^r^_*q*_ is derived from the corresponding ff14SB bonded parameter reduced by 17% to aid higher flexibility (Fig. 1A).

For *γ*_m_ < 1, the effective strength of the intramolecular solute potential is reduced, producing the same physical effect as in REST2, *i.e.*, an elevated effective solute temperature while the solvent remains unperturbed. Since *U*^r^ scales with *η*_m_ and *η*_1_ = 0, the reference replica 1 satisfies *U*^r^_1_ = 0, preserving the target Boltzmann ensemble. When *γ*_m_ decreases along the replica ladder, intramolecular interactions are softened while harmonic stabilization of bonds and angles (*η*_m_) increases, maintaining structural integrity under strong solute tempering (Fig. S1). All replicas are propagated at the same thermostat temperature *T*, and exchanges are accepted according to the standard Metropolis criterion for Hamiltonian replica exchange.^67^ The corresponding exchange exponent is given by,4
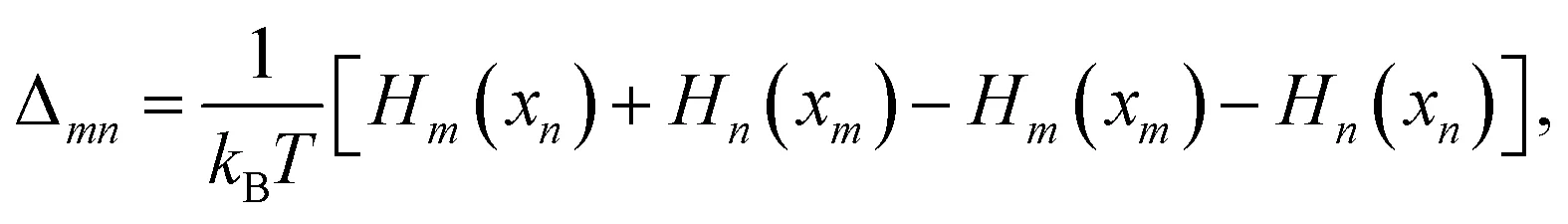
where *k*_B_ is the Boltzmann constant. The acceptance probability min(1, e^−Δ_*mn*_^) ensures detailed balance with respect to the target ensemble.

The method gREST^68^ has been developed using classical force fields to allow selected molecular regions to be scaled independently. Although selective scaling of a subset of atoms would in principle be possible with AMP/MM, the tempered region is tied to the ML region in the current implementation. Since the AMP-BMS model can describe an entire protein within the ML zone,^35^ the method remains applicable to protein systems, although selective tempering may improve its efficiency for very large systems.

Importantly, the presented REST2-like framework is not restricted to the AMP family of models and can, in principle, be generalized to other atomistic NNPs and electrostatic-embedding ML/MM approaches that allow for similar partitioning of the Hamiltonian. Implementations of REST2 are also available in other widely used MD engines such as AMBER^69^ and GROMACS,^70^ supporting the portability of the present approach. These MD engines also provide flexible definitions of harmonic bond and angle restraints.

The introduction of adaptive harmonic restraints stabilizes replicas with high scaling factors without perturbing the target ensemble, ensuring numerical robustness even under strong tempering conditions. We also want to stress that these restraints can be applied selectively to specific bonds or atoms, therefore, reactive regions of the system can remain fully unrestrained, enabling also the study of processes that involve bond-breaking or bond-formation events.

### Enhanced conformational sampling of small peptides

For all AMP/MM simulations, we used the global AMP-BMS model^35^ trained on the BMS25 dataset^65^ without any fine-tuning. This choice is justified by the model's intended applicability to peptides, proteins, and small organic molecules. As shown in Table S1 in the SI, AMP-BMS yields energies and forces consistent with the underlying QM/MM reference calculations at the target level of theory.^71–79^

#### Alanine dipeptide

We simulated alanine dipeptide to evaluate the ability of the algorithm to enhance the exploration of conformational space described by the backbone *ϕ* and *ψ* dihedral angles shown in Fig. 2A. The system was simulated for 20 ns using the REST2 protocol with eight replicas. The scaling factors were geometrically distributed between 1 and 0.125 to maximize potential-energy overlap between highly-scaled neighboring replicas (see Fig. S1 for an illustrative example). This setup resulted in an average exchange acceptance rate of 17%, consistent with optimal replica-exchange efficiency.^80^ We first validated the correct behavior of the algorithm by performing a round-trip analysis (details provided in the SI and in Fig. S3). As shown in Fig. 2B, the coordinates associated with walker 1 undergo a high number of round trips across the replica ladder, indicating efficient mixing between replicas. The stability of the sampled conformational ensemble was further assessed using customTABS.^81^ The time-dependent populations of the ten most populated conformational classes determined by customTABS are reported in Fig. S11. When analyzing the sampled conformations using Ramachandran plots for each replica (Fig. 2C), we found that higher replicas exhibit progressively broader coverage of the *ϕ* and *ψ* dihedral-angle space, indicating effective reduction of intramolecular energy barriers under REST2 scaling. This enhanced exploration enables sampling of high-energy conformations that are rarely visited in the unscaled ensemble. Through replica exchange, these configurations are subsequently reweighted under neighboring Hamiltonians and contribute to the canonical distribution of the lowest replica 1.

**Fig. 2:**
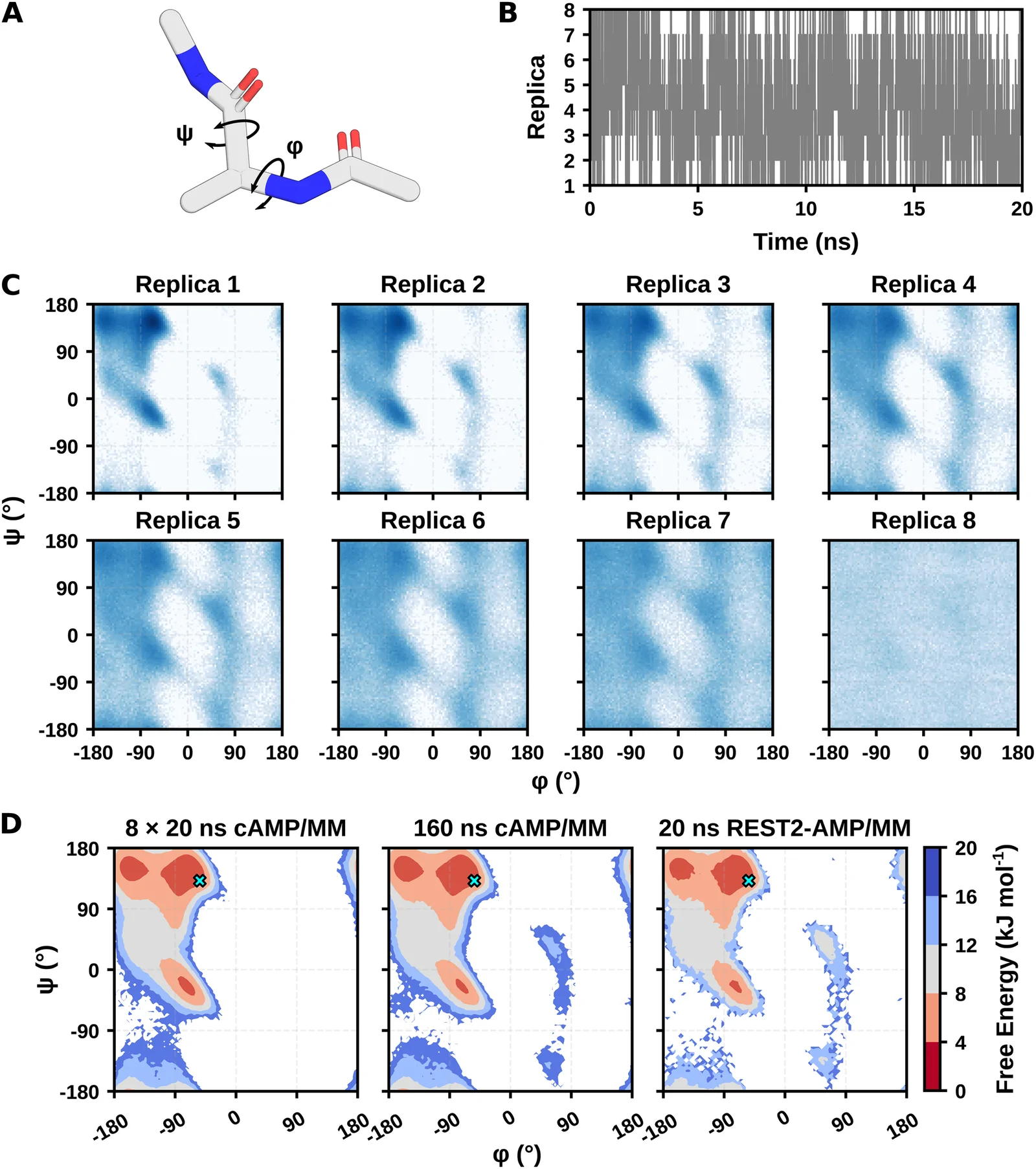
REST2-AMP/MM simulation of alanine dipeptide. (A) 3D representation. (B) Replica index as a function of simulation time for walker 1. (C) Ramachandran (*ϕ*, *ψ*) distributions sampled by each replica. (D) Free-energy landscapes projected onto the Ramachandran (*ϕ*, *ψ*) space for cAMP/MM simulations consisting of eight independent trajectories of 20 ns each (left), a single cAMP/MM simulation of 160 ns (middle), and REST2–AMP/MM simulations with a total simulation time of 20 ns (right). The REST2 simulation was carried out for 20 ns per replica using eight replicas, corresponding to a cumulative simulation time of 160 ns across the replica ensemble. Thus, all three protocols correspond to the same cumulative simulation time of 160 ns. The cyan cross represents the starting point of the simulations.

To assess whether REST2 improves the exploration of alanine dipeptide's configuration space, we compared REST2-AMP/MM performance against two reference simulation protocols. First, we carried out a single conventional AMP/MM (cAMP/MM) simulation of 160 ns. Second, we compared the REST2 simulation with eight 20 ns cAMP/MM trajectory segments, consisting of the first 20 ns of the 160 ns trajectory and seven independent 20 ns trajectories generated with different initial velocities, yielding the same cumulative simulation time of 160 ns. These simulations enable a direct comparison between REST2-enhanced and conventional simulations. The resulting free-energy landscapes are summarized in Fig. 2D. Both set-ups of cAMP/MM simulations are able to sample the main conformational states of alanine dipeptide, such as the *P*_II_ and *α*_R_ states,^34^ which populate the left quadrants of the Ramachandran plot.^82^ The 160 ns cAMP/MM protocol also accesses the right quadrant of the Ramachandran plot, although only after 150 ns (Fig. S4 and Fig. S10). In contrast, REST2 achieves a markedly more efficient exploration of this region, sampling it already within 1 ns. As a result, REST2 more readily populates relevant conformational states, including *α*_L_, *C*7_ax_, and *α*_D_,^34^ in agreement with the ranking expected from Raman optical activity experiments^82^ and previous force-field-based simulation studies.^47^

These results indicate that the improved sampling efficiency does not compromise the accuracy of the underlying potential, and further highlight the ability of the global AMP-BMS model^35^ to accurately reproduce the conformational landscape without fine-tuning.

#### Cyclo-(Pro–Ser–Leu–Asp–Val)

To further validate the enhanced-sampling protocol, we performed simulations of cyclo-(Pro–Ser–Leu–Asp–Val) in both the *cis*- and *trans*-states (Fig. 3A,B), defined according to the Val-Pro *ω* dihedral-angle as described previously.^83^ The *cis*- and *trans*-systems were each simulated for 50 ns with cAMP/MM and REST2-AMP/MM, respectively, employing eight replicas with scaling factors linearly distributed between 1.0 and 0.5. The stability of the sampled conformational ensemble was further assessed using customTABS^81^ (see Fig. S11). To assess the structural accuracy of the simulations, we compared computed proton–proton NOE distances with experimental NMR data acquired in aqueous solution.^84^ For this purpose, we calculated *r*^−6^-averaged proton–proton distances from the lowest replica and subtracted the experimental NOE upper bounds (Fig. 3C) to determine the violations.

**Fig. 3:**
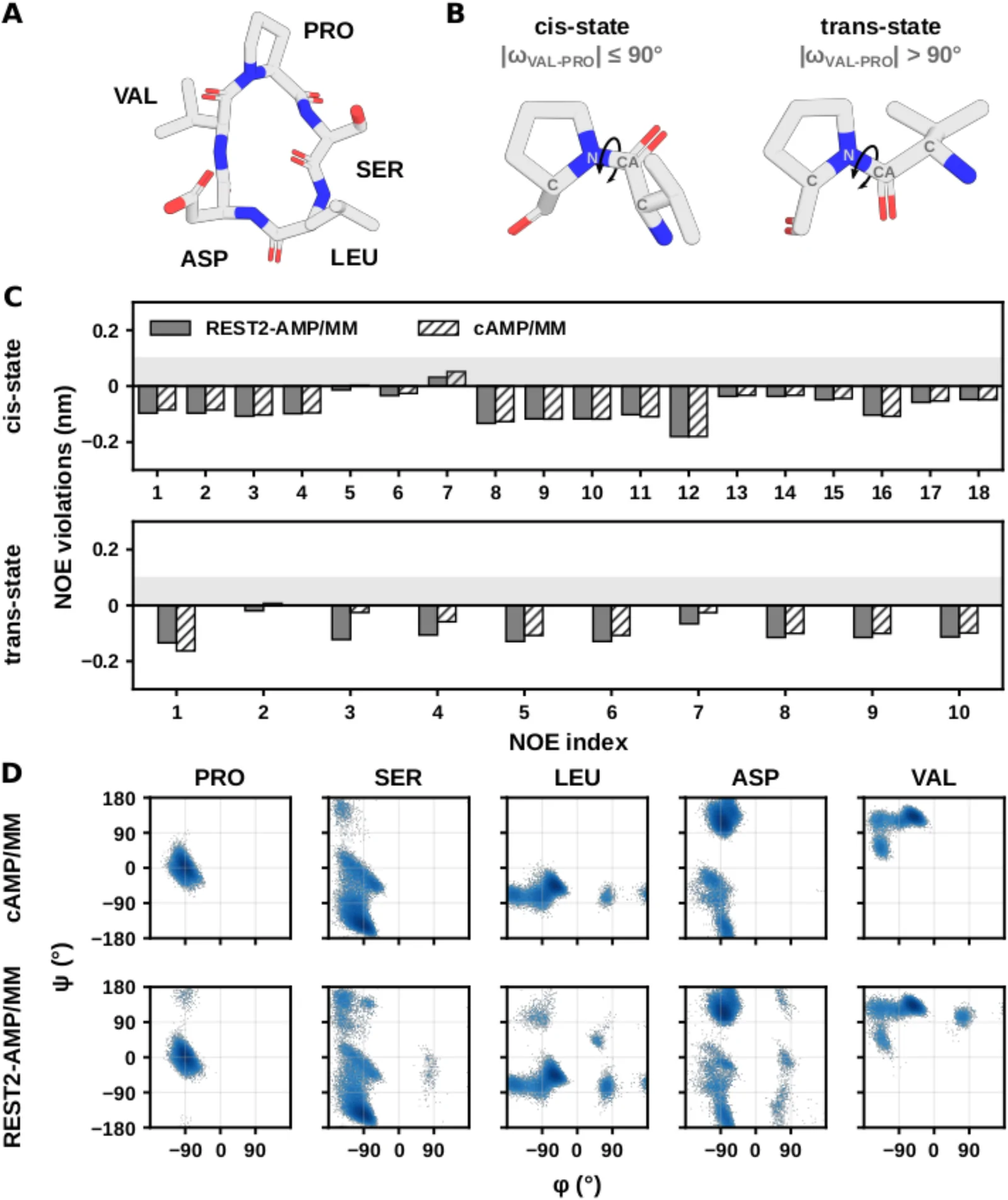
Conformational analysis of the cyclic pentapeptide and comparison with NOE restraints. (A) Representative structure of cyclo-(Pro–Ser–Leu–Asp–Val) with residues labeled. (B) Definition of the *cis*- and *trans*-states of the Val–Pro peptide bond based on the *ω* dihedral angle (*cis*: |*ω*_VAL–PRO_| ≤ 90°; trans: |*ω*_VAL–PRO_| > 90°). (C) Violations of NOE upper distance bounds for structural ensembles calculated in the *cis*-state (top) and *trans*-state (bottom). Bars indicate the magnitude of violation (in nm) for each NOE index; the shaded region denotes the acceptable tolerance range. (D) Ramachandran (*ϕ*, *ψ*) distributions for each residue from cAMP/MM (top row) and REST2-AMP/MM (bottom row) simulations.

Overall, both conventional and enhanced AMP/MM simulations show excellent agreement with the experimental constraints. In the *cis*-state simulations, only one NOE proton pair exceeds the experimental upper bound, corresponding to the backbone distance between Asp and Leu. However, the deviation is minimal (<0.05 nm). In the *trans*-state REST2 simulations, all NOE distances satisfy the experimental upper bounds. To evaluate the extent of sampling enhancement, we analyzed the Ramachandran dihedral angle distributions of each residue in the cyclic pentapeptide and compared the conformational space explored in cAMP/MM and REST2-AMP/MM simulations. As shown in Fig. 3D for the *cis*-state, REST2-AMP/MM significantly broadens the sampled (*ϕ*, *ψ*) space relative to conventional simulations, while preserving the dominant conformational regions consistent with the experimental restraints. The enhanced sampling enables transitions between conformational basins that are only sparsely populated in cAMP/MM simulations, indicating improved exploration of backbone flexibility. A similar qualitative behavior is observed for the *trans*-state simulations (Fig. S5). Note that the *cis*/*trans*-isomerization itself is associated with such a high energy barrier that it was not observed even with the REST2-like enhanced sampling in our set-up.

### Partial folding of peptide systems

#### Polyproline

Polyproline chains adopt a characteristic *P*_II_ helical secondary structure, defined by backbone dihedral angles of approximately (*ϕ*, *ψ*) ≈ (−75°, 150°) and trans peptide-bond isomers.^85^ In this study, we selected a peptide composed of 13 prolines (Fig. 4A). We first simulated polyproline in the stable *P*_II_ conformation using REST2–AMP/MM for 25 ns to verify that AMP-BMS reproduces the expected conformational landscape reported in experimental and computational studies.^85,86^ We employed 12 replicas with scaling factors linearly distributed between 1 and 0.33. Projection of the resulting trajectories onto the backbone (*ϕ*, *ψ*) dihedral-angle space confirmed stable population of the *P*_II_ minimum across replicas (Fig. S6).

**Fig. 4:**
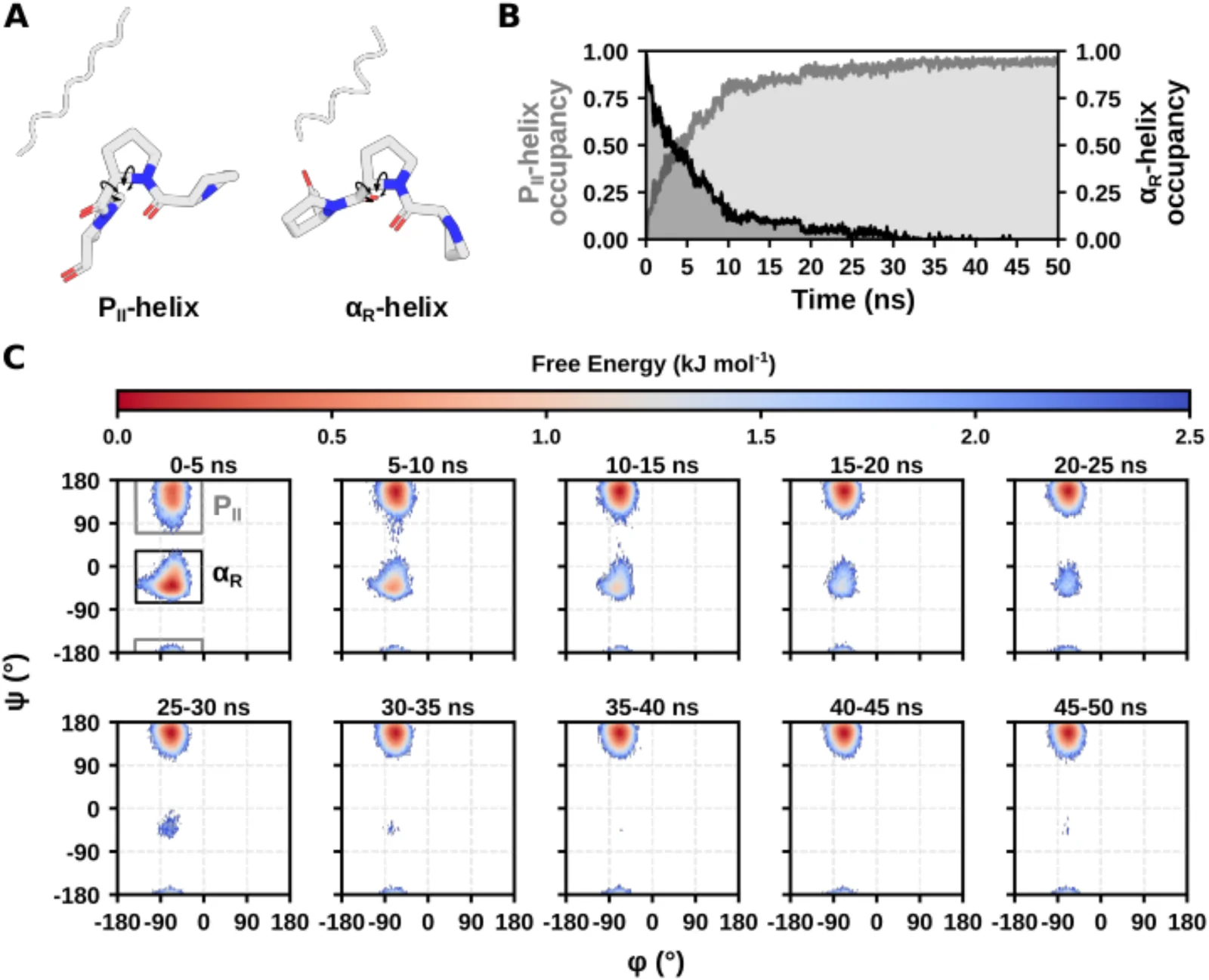
REST2-AMP/MM simulations of polyproline. (A) Representative structures of polyproline in native *P*_II_-helix and the misfolded *α*_R_-helix conformations. (B) Time evolution of the conformational populations during the simulation: *α*_R_-helix (black) and *P*_II_-helix (grey). (C) Time-resolved Ramachandran (*ϕ*, *ψ*) distributions of replica 1 divided into ten consecutive trajectory segments.

Next, we intentionally modeled the peptide in an *α*-helical conformation (Fig. 4A) to evaluate the structure-refinement capability of our protocol and test whether REST2–AMP/MM can efficiently explore conformational space and recover the thermodynamically preferred structure. After building the *α*-helix model in PyMOL/3.1.6.1,^87^ we applied the same REST2 set-up for 50 ns. As expected, higher replicas sampled a substantially broader region of (*ϕ*, *ψ*) space, crossing energetic barriers that remain inaccessible in the unscaled replica (Fig. S7). To quantify the structural transition, we monitored the occupancy of two Ramachandran basins over time. The upper basin, corresponding to the *P*_II_ helix, comprises configurations with *ϕ* ∈ [−90°, – 40°] and *ψ* ∈ [100°, 180°]. The lower basin, associated with the *α*_R_ region, includes *ϕ* ∈ [−110°, – 40°] and *ψ* ∈ [−90°, 20°]. We defined both basins as rectangular regions in (*ϕ*, *ψ*) space. As shown in Fig. 4B, the system initially resides entirely in the *α*_R_-helical state (population = 1.00), while the *P*_II_ population remains near zero. Within the first 15 ns, the *α*_R_ population decreases sharply as the structure converts and the *P*_II_ population increases at a comparable rate. By 20 ns, the *P*_II_ population reaches a stable plateau, remaining close to 0.90 for the remainder of the simulation, whereas the *α*_R_ population remains near zero. These results demonstrate an almost complete structural conversion and confirm the *P*_II_ conformation as the energetically favored state under the simulated conditions.

Time-resolved Ramachandran plots (Fig. 4C) provide a complementary view of this transition. The initial chunk (0–5 ns) displays two high-density regions centered at (*ϕ*, *ψ*) ≈ (−60°, – 40°) for the *α*_R_ helix and (−75°, 150°) for the *P*_II_ helix. As the simulation progresses, the *α*_R_ cluster steadily loses intensity, while the *P*_II_ region gains population. From chunk 8 (35–40 ns) onward, the *α*_R_ basin nearly disappears and the *P*_II_ region dominates the distribution, confirming the conversion from an initially *α*_R_-helical ensemble to a stable *P*_II_ state.

These results indicate that REST2–AMP/MM efficiently enhances the exploration of the conformational landscape and enables the recovery of the thermodynamically preferred *P*_II_ helix from a misfolded *α*-helical state. These results also affirm the ability of AMP-BMS itself to properly describe the dynamics of peptidic systems.

#### Trp-cage mini-protein

To test our approach on a more challenging application, we intentionally modeled the *α*-helical segment of the Trp-cage mini-protein (residues 1–10) as a coil. This setup provides a test to assess whether REST2-AMP/MM enables recovery of the native structure through partial refolding toward the experimentally determined state.^88^ A similar protocol based on classical force fields has been previously applied.^48,89^ As for polyproline, we first validated the stability of the native fold using both cAMP/MM and REST2-AMP/MM. Simulations were initiated from the experimental solution NMR structure (PDB ID: 1L2Y^88^) and simulated for 50 ns. To assess structural fidelity, NOE-derived proton–proton distances were calculated from the generated ensembles and compared with experimental reference values.^88^ Both simulation protocols showed good agreement with experimental NOE restraints (Fig. 5A). REST2-AMP/MM and cAMP/MM violated only three and two NOE distances, respectively, with only one violation exceeding the NMR upper bound by more than 0.1 nm. These results confirm that AMP-BMS is able to preserve the stability of the folded Trp-cage structure in agreement with experiment.

**Fig. 5:**
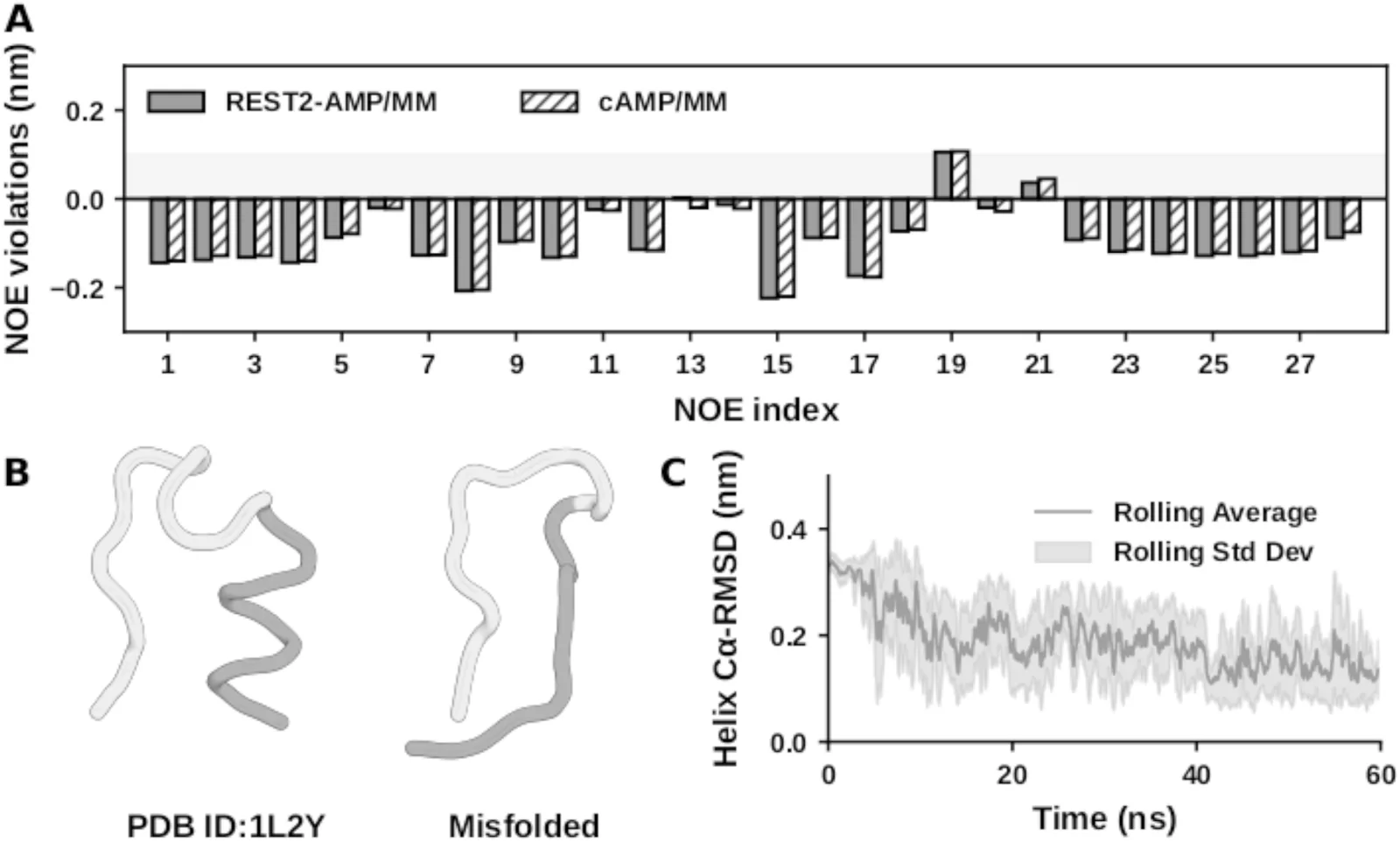
REST2-AMP/MM simulations of Trp-cage mini-protein. (A) NOE distance violations of cAMP/MM (white) and REST2-AMP/MM (gray) simulations initiated from the experimental NMR structure. The shaded region indicates the 0.1 nm tolerance range. (B) Folded (PDB ID: 1L2Y^88^) and partially misfolded conformations used as starting coordinates. (C) Time evolution of the helix C_α_-RMSD with respect to the reference structure in the simulation started from the misfolded conformation, shown as the rolling average (dark grey) with the corresponding rolling standard deviation (light grey).

Next, we constructed a modified Trp-cage structure in which the native *α*-helix was replaced by a coil conformation (Fig. 5B). Starting from this non-native configuration, we performed 60 ns REST2-AMP/MM simulations to determine whether the protocol enables recovery of the correct helical fold. The refolding process was monitored using C_α_ RMSD calculations of the seven central helix residues (residues 3–9). Simulations initiated from the misfolded configuration showed a rapid structural rearrangement during the early stages of the trajectory. As shown in Fig. 5C, the C_α_ RMSD decreases substantially within the first few nanoseconds, indicating a transition toward conformations closer to the native helical structure. Following this initial rearrangement, the RMSD stabilizes around ∼0.15–0.20 nm, suggesting that the central segment adopts and maintains a conformation consistent with the native helix for the remainder of the simulation.

These results highlight the capability of REST2–AMP/MM to perform high-accuracy structure refinement, which might be valuable for refining structures generated at a lower level of theory (*e.g.*, classical MD simulations) or directly inferred from generative deep learning algorithms (*e.g.* AlphaFold3 ^90^ or Boltz-2 ^91^). We note that for both polyproline and Trp-cage, the observed plateaus indicate stability of the monitored structural observables over the simulated timescale, but should not be interpreted as convergence of the complete thermodynamic ensemble.

### Reaction-coordinate-free folding simulations

A key advantage of REST2 lies in its ability to enhance the sampling of the free-energy landscape without the need to predefine a reaction coordinate, thereby eliminating the requirement for prior knowledge of the folding pathway. This capability has been demonstrated in previous force-field-based REST2 simulations.^89^ Building on this, we conducted reaction-coordinate-free folding ML/MM simulations of the mini-protein chignolin and its faster-folding mutant CLN025.

#### CLN025

Fig. 6A shows the folded structure of CLN025 obtained from the solution NMR ensemble (PDB ID: 2RVD^92^) together with the fully extended conformation used to initiate folding simulations. To verify that the folded structure is stable with AMP-BMS, we first simulated CLN025 starting from the NMR structure using 15 ns REST2 simulations with 12 replicas and scaling factors linearly distributed between 1 and 0.5. The trajectories were projected onto the 2D space defined by the radius of gyration (*R*_g_) and the intramolecular distance RMSD_dist_ with respect to the NMR structure (see eqn (5) and Fig. S8). From the free-energy landscape of replica 1, we located the most populated region in the (*R*_g_, RMSD_dist_) space and defined this region as the reference folded structure.

**Fig. 6:**
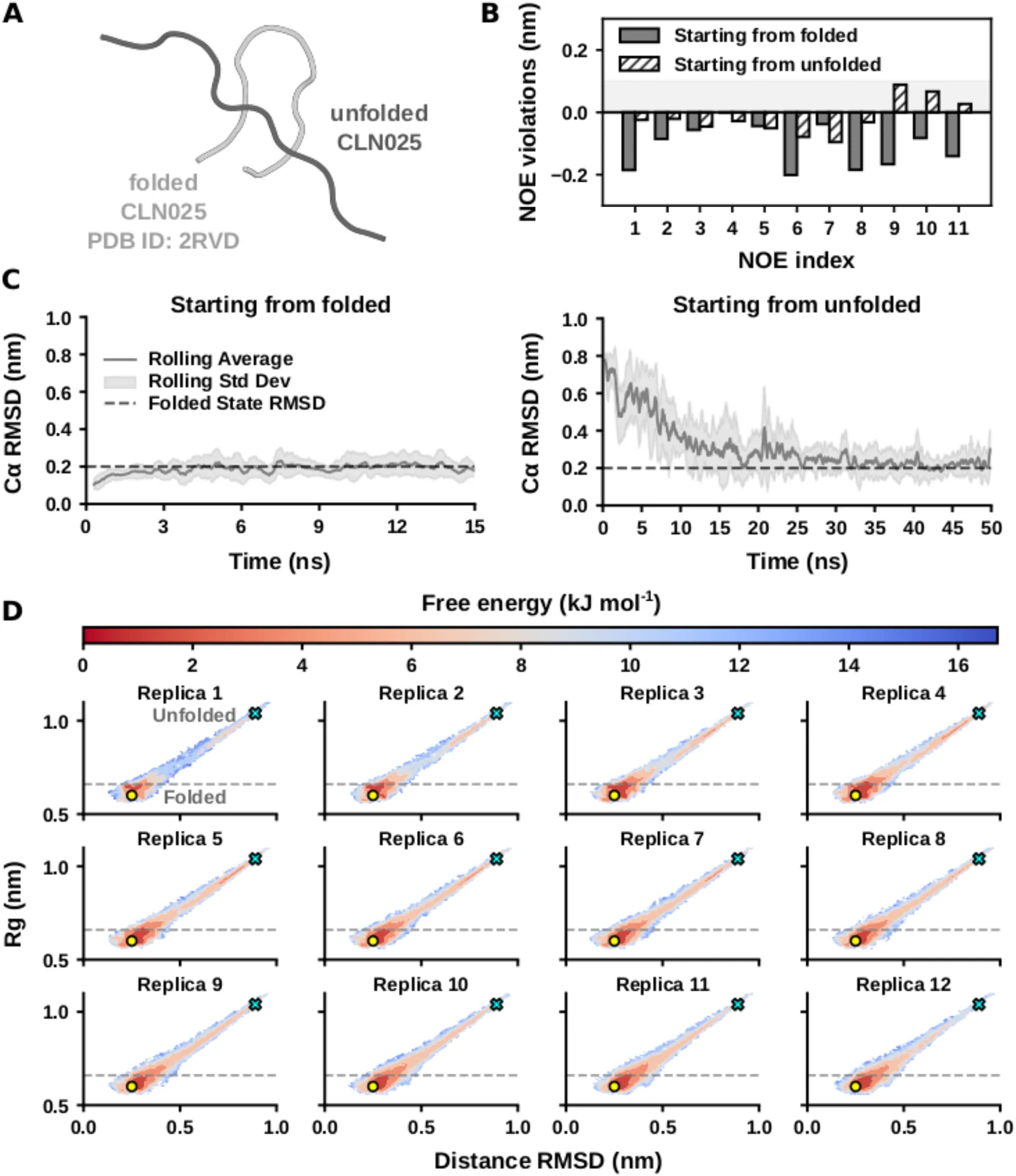
Reaction-coordinate-free REST2 simulations of CLN025. (A) Folded NMR solution structure (PDB ID: 2RVD^92^) and a fully extended conformation used as starting coordinates. (B) Violations of the computed *r*^−6^-averaged NOE distances with respect to the experimental NOE upper bounds for REST2 simulations initiated from the folded (dark gray bars) and unfolded (white striped bars) conformations. For the former, the full trajectory was used, whereas the last 50% of replica 1 was considered for the latter. (C) Time evolution of the C_α_-RMSD of replica 1 with respect to the NMR structure for the REST2 simulation starting from the folded (left) and extended (right) conformations. (D) Free-energy landscapes projected onto the (*R*_g_, RMSD_dist_) space for the 12 replicas (kJ mol^−1^) starting from the extended conformation. The yellow marker denotes the reference folded structure identified in the REST2 simulation started from the folded structure; the dashed line (*R*_g_ ≈ 0.65 nm) marks the threshold for compact conformations. The cyan cross represents the starting point.

We compared the simulated conformations against experimental NOE distance restraints derived from NMR measurements.^92^ The distances from the entire trajectory were *r*^−6^-averaged and the experimental NOE upper bounds were subtracted. The atom pairs involving the first and last two residues were excluded to focus on interactions defining the folded core. The resulting NOE violations are shown as dark gray bars in Fig. 6B. All distance differences are below 0 nm, indicating good agreement between the simulated ensemble and experimental NMR data. Note that the C_α_-RMSD of the folded conformation of replica 1 is at 0.2 nm with respect to the NMR structure (left panel in Fig. 6C).

Next, we characterized the folding behavior of CLN025 by starting the REST2 simulation from an extended conformation (Fig. 6A) and monitoring the time evolution of the C_α_-RMSD of replica 1 with respect to the NMR structure (right panel in Fig. 6C). Simulations begin with RMSD values above 0.8 nm and decrease steadily over time. After approximately 30 ns, the RMSD reaches a plateau at ∼0.2 nm and remains stable around this value, indicating that the system reaches the same folded basin identified from the native-state simulations. This can also be seen in the NOE violations calculated from the last 50% of the trajectory of replica 1 (white striped bars in Fig. 6B). Most distance differences fall within the ±0.1 nm interval and none of the analyzed pairs exceed the +0.1 nm threshold, which shows agreement with the experimental NMR restraints. The overall sampling behavior in the 50 ns REST2 simulation is illustrated in Fig. 6D, which shows the free-energy landscapes in the (*R*_g_, RMSD_dist_) space for all 12 replicas. In every case, the highest-density regions cluster around *R*_g_ ≈ 0.6 nm and RMSD_dist_ ≈ 0.25 nm, corresponding to the folded reference structure (yellow marker). The dashed horizontal line at *R*_g_ ≈ 0.65 nm highlights the compact-state threshold. Across all replicas, the dominant density consistently overlaps with the native basin.

#### Chignolin

We next applied the same simulation protocol to the slower-folding chignolin^93,94^ (*i.e.*, 12 replicas with scaling factors linearly distributed between 1 and 0.5), starting from either the NMR solution structure (PDB ID: 1UAO^95^) or the extended conformation. Fig. 7A shows the time evolution of the C_α_-RMSD with respect to the NMR structure for replica 1. When starting from the folded conformation, chignolin remains folded with an RMSD value around ∼0.2 nm with respect to the NMR structure (left panel), as seen previously for CLN025. Based on this simulation, the reference folded state was identified as the most populated region of replica 1 when projected onto the 2D space defined by *R*_g_ and the intramolecular distance RMSD_dist_ (see eqn (5) and Fig. S9).

**Fig. 7:**
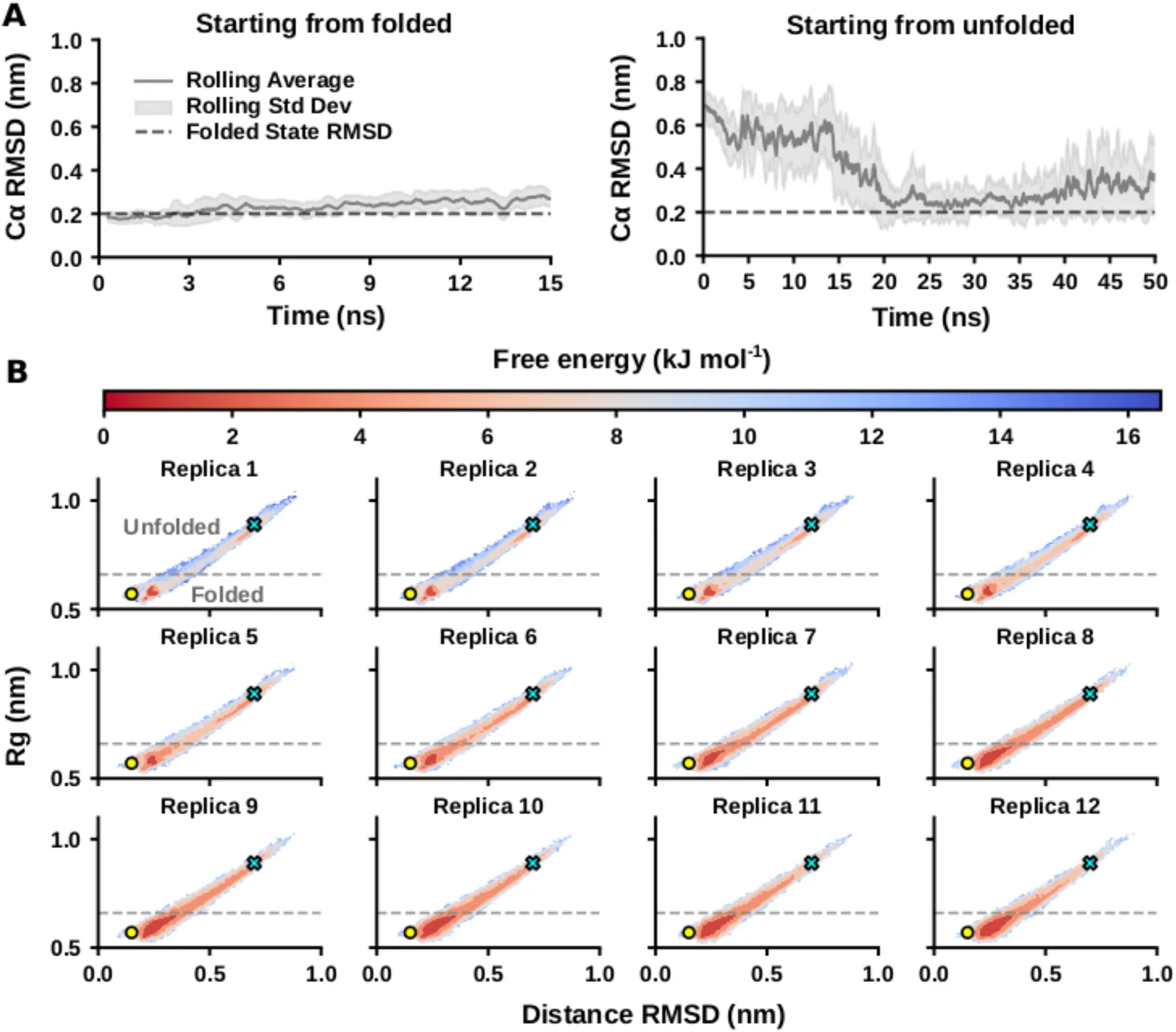
Reaction-coordinate-free REST2 simulations of chignolin. (A) Time evolution of the C_α_-RMSD of replica 1 with respect to the folded NMR structure (PDB ID: 1UAO^95^) for simulations starting from the folded (left) and fully extended (right) conformations. (B) Free-energy landscapes projected onto the (*R*_g_, RMSD_dist_) space for the 12 replicas (kJ mol^−1^) starting from the extended conformation. The yellow marker denotes the reference folded structure identified in the REST2 simulation started from the folded structure; the dashed line (*R*_g_ ≈ 0.65 nm) indicates the threshold for compact conformations. The cyan cross represents the starting point.

When starting the REST2 simulation from the extended conformation, the C_α_-RMSD begins above 0.6 nm and shows a progressive decrease over time (right panel in Fig. 7A). After approximately 20–25 ns, the RMSD reaches values of ∼0.2–0.3 nm and fluctuates around this level for the remainder of the trajectory, indicating that the system reached the folded basin. Fig. 7B shows the free-energy landscapes of the 12 replicas in the (*R*_g_, RMSD_dist_) space. Across all replicas, the highest-density regions cluster around *R*_g_ ≈ 0.6 nm and RMSD ≈ 0.15 nm, corresponding to the folded reference structure (yellow marker). The dashed horizontal line at *R*_g_ ≈ 0.65 nm highlights the compact-state threshold.

We want to highlight that while the folding dynamics of chignolin and/or CLN025 have been previously studied using NNPs in conjunction with reaction-coordinate-based enhanced-sampling algorithms,^23^ the REST2-like framework bypasses the requirement for predefined reaction coordinates and explores the conformational energy landscape autonomously, which means that the folded state does not have to be known beforehand. This makes the framework a promising tool for the unbiased investigation of unknown free-energy landscapes in complex peptidic systems. We emphasize that these simulations demonstrate recovery of the experimentally characterized folded conformations from extended starting structures, but do not establish equilibrium folding populations or folding–unfolding reversibility. Demonstrating thermodynamic convergence would require substantially longer simulations with multiple independent folding–unfolding transitions.

## Conclusions

A general and transferable REST2-like framework for electrostatically embedded ML/MM simulations has been presented and combined with the global AMPv3-EE-BMS25 model (short AMP-BMS). By exploiting the decomposition of the AMP/MM Hamiltonian into solute–solute, solute–solvent, and solvent–solvent contributions, REST2-like scaling of the ML-described region and its interactions with the environment is enabled without altering the physical temperature of the solvent. This formulation preserves the theoretical basis of classical REST2 while extending its applicability to modern multiscale NNPs.

The resulting REST2-AMP/MM approach was validated on peptidic systems of increasing complexity. For alanine dipeptide, the canonical conformational states were reproduced and higher-energy basins were sampled more efficiently than in conventional AMP/MM simulations, without loss of accuracy. Agreement with experimental NOE distances for the cyclic pentapeptide, Trp-cage mini-protein, and CLN025, together with recovery of the characteristic *P*_II_ secondary structure of polyproline, further supports the ability of AMP-BMS to accurately describe the dynamics of peptidic systems. When simulations were initiated from partially misfolded structures, efficient exploration of the relevant conformational space and recovery of experimentally consistent structural motifs were observed for both polyproline and Trp-cage mini-protein. These findings indicate the potential of REST2-AMP/MM for high-accuracy structure refinement, for example, for structures generated at lower levels of theory or by modern deep learning models for structure prediction. Finally, reaction-coordinate-free folding of chignolin and CLN025 from extended conformations was achieved, demonstrating that conformational landscapes can be explored autonomously without predefined reaction coordinates.

We focused in the present study on peptidic systems because the BMS25 training set underlying the global AMP-BMS model is centered on peptides and proteins. However, enhanced-sampling ML/MM approaches are expected to be especially promising for non-peptidic systems, for which the classical force fields may be less reliably parameterized or may fail to capture important electronic effects such as polarization and conjugation.

Beyond peptidic systems, the present framework should extend directly to enzymatic reactions, provided that atoms involved in bond breaking or bond formation are excluded from the harmonic restraints. Since AMP-BMS can, in principle, describe an entire protein within the ML zone, defining solute and solvent regions for an enzyme is, from a practical standpoint, no different from doing so for the peptidic systems studied here. However, tempering the full protein may become inefficient for large systems because of the larger number of scaled degrees of freedom and the associated computational cost. gREST addresses this issue by restricting tempering to selected regions, and a similar selective-scaling strategy would be a useful extension of the present framework. Intrinsically disordered proteins (IDPs) are another promising application: AMP-BMS was previously shown to qualitatively reproduce melting-curve trends for IDPs, but the limited sampling used there (three 100 ns replicates) led to large uncertainties in the fitted curves. REST2-AMP/MM could enhance sampling of the folded/unfolded ensemble and improve the accuracy of such predictions.

A general challenge of NNPs lies in their dependence on the training set. Simulating molecules or conformational states that are underrepresented, or entirely absent, from the training data may lead to reduced accuracy or instabilities during the simulation. In addition, although AMP-BMS significantly accelerates simulations compared to CPU-based DFT calculations, NNPs remain computationally more demanding than classical force fields. While they offer substantially higher accuracy, reaching biologically relevant timescales may still require considerable computational resources, including multiple GPUs, a significant amount of V-RAM and extended wall-clock times.

Nevertheless, the present results establish REST2-AMP/MM as a transferable route toward enhanced sampling with near-DFT accuracy and suggest that further developments along this direction could broaden the applicability of ML/MM simulations to increasingly complex biomolecular and chemically diverse systems.

## Methods

### Preparation of the simulation systems

All systems were solvated in TIP4P-FB water^96^ using OpenMM/8.1.2 (ref. 97) with a padding distance of 1.5 nm. No counter-ions were added to the simulation boxes. Lennard-Jones (LJ) parameters for particles within the ML region, used to describe the LJ contribution to the ML/MM interaction, were assigned using the AMBER ff14SB force field.^66^ The preparation of the solute structures prior to solvation is described below for each system.

#### Alanine dipeptide

The atomic coordinates of alanine dipeptide were taken from the alanine dipeptide toy systems distributed with OpenMM/8.1.2.^97^

#### Cyclo-(Pro–Ser–Leu–Asp–Val)

The system was modeled in both the *cis*-proline and *trans*-proline states, depending on the torsion angle described in the experimental work.^84^ Coordinates of the *cis*-state were generated using the Boltz-2 server provided by NVIDIA,^91^ employing standard settings and explicitly specifying that the system is a cyclic peptide. For the *trans*-state, the molecule was embedded using RDKit/2023.3.1,^98^ and a conformer was generated using the ETKDGv3.^99^ The conformer was subsequently minimized in OpenMM/8.1.2 (ref. 97) for a maximum of 2000 iterations. During minimization, harmonic torsion restraints were applied to the atoms defining the relevant dihedral angle, using a force constant of 10 000 kJ mol^−1^ rad^−2^, in order to maintain the torsion angle around 110° (|*ω*_VAL–PRO_| > 90°).

#### Polyproline

Coordinates were generated using the PyMOL/3.1.6.1 builder tool.^87^ For the misfolded conformation, the chain was assembled by setting the residue arrangement to an *α*-helix. The resulting structure was then processed with the *pdb4amber* utility from AmberTools^100^ to make it compatible with the AMBER force field. The system was also modeled in the correctly folded conformation. As the *P*_II_ secondary structure is not directly available in the builder module in PyMOL/3.1.6.1,^87^ residues 1 to 13 were first constructed in an antiparallel β-sheet secondary structure and then processed as described above. After the equilibration phase, the structure had relaxed into a *P*_II_ conformation.

#### Trp-cage mini-protein

Coordinates of the folded conformation were taken from the PDB^101^ (PDB ID: 1L2Y^88^). N-terminal acetyl (ACE) and C-terminal *N*-methylamine (NME) capping groups were added using PyMOL/3.1.6.1.^87^ Protonation states were assigned using the H++ web server.^102^ The resulting structure was subsequently processed with the *pdb4amber* utility from AmberTools.^100^ The system was also modeled in a misfolded conformation. For this, we used a similar procedure as for polyproline. Residues 1 to 10 were first constructed in an antiparallel β-sheet secondary structure using the builder module in PyMOL/3.1.6.1,^87^ and then protonated and processed as described above. After the equilibration phase, the first 10 residues had relaxed into a coil conformation.

#### CLN025

Coordinates of the folded conformation were taken from the PDB (PDB ID: 2RVD^92^) and protonation states were assigned using the H++ web server.^102^ The C- and N-termini were charged and not capped. We subsequently processed the resulting structure with the *pdb4amber* utility from AmberTools.^100^ The system was also modeled in an extended conformation using *tleap*.^100^ The system was protonated and processed as described above.

#### Chignolin

Coordinates of the folded conformation were taken from the PDB (PDB ID: 1UAO^95^) and protonation states were assigned with the H++ web server.^102^ The C- and N-termini were charged and not capped. We processed the resulting structure with the *pdb4amber* utility from AmberTools.^100^ Chignolin was also modeled in an extended conformation using *tleap*.^100^ The system was protonated and processed as described above.

### Conventional AMP/MM simulations

We performed cAMP/MM MD simulations of alanine dipeptide, *cis*-cyclo-(Pro–Ser–Leu–Asp–Val), *trans*-cyclo-(Pro–Ser–Leu–Asp–Val), and the Trp-cage mini-protein using OpenMM/8.1.2 (ref. 97) with OpenMM–PyTorch/1.4.^97,103^ Bonds involving MM water molecules were constrained using the SETTLE algorithm.^104^ A nonbonded cutoff of *r*_MM/MM_ = 0.9 nm was employed and long-range electrostatic interactions were treated using the shifted reaction field method.^105^ A long-range correction for the LJ potential^106^ was applied. Center of mass translation was removed every step. All simulations were performed using a Langevin middle integrator with a timestep of Δ*t* = 1 fs and a friction coefficient of 1 ps^−1^ at a reference temperature of 298.15 K.^107^ In NPT simulations, the target pressure of 1 bar was maintained using a Monte Carlo barostat^108^ with a trial frequency of *n* = 100. The AMPv3-EE-BMS25 model^35^ with the polarization scaling scheme (*λ*_pol_ = 0.35) was used for all AMP/MM simulations. All AMP-BMS model parameters as well as the model weights are identical to the original publication of the model.^35^

The systems were first energy-minimized until the maximum force on any atom was below the default tolerance of 10 kJ mol^−1^ nm^−1^, followed by equilibration in the NVT ensemble for 100 ps. Subsequently, the systems were equilibrated in the NPT ensemble for 1 ns to reach the target pressure. Production simulations of the systems were carried out in the NPT ensemble.

In total, we performed eight independent simulations of alanine dipeptide, consisting of one trajectory of 160 ns and seven trajectories of 20 ns each. For *cis*-cyclo-(Pro–Ser–Leu–Asp–Val), *trans*-cyclo-(Pro–Ser–Leu–Asp–Val) and the Trp-cage mini-protein, we carried out a single 50 ns simulation.

### REST2-AMP/MM simulations

For all systems, simulations were performed using the same MD settings as described in Section “Conventional AMP/MM simulations”, with exchange attempts between adjacent replicas carried out every 100 integration steps. The optimal choice of REST2 parameters is inherently system dependent and therefore must be established empirically. In particular, parameter selection requires balancing a sufficiently high exchange acceptance rate with maintaining the overall stability of the simulations. The REST2 parameters employed for each system are summarized in Table 1, and the corresponding wall-clock performance is reported in Table S2.

**Table 1:** Summary of REST2-AMP/MM parameters. (f) Starting from folded conformation; (u) starting from unfolded (fully extended) or misfolded conformation

System	Time (ns)	Replicas/scaling
Alanine dipeptide	20	8/geometric (1–0.125)
*cis*-Cyclo-(Pro–Ser–Leu–Asp–Val)	50	8/linear (1–0.5)
*trans*-Cyclo-(Pro–Ser–Leu–Asp–Val)	50	8/linear (1–0.5)
Polyproline (f)	25	12/linear (1–0.33)
Polyproline (u)	50	12/linear (1–0.33)
Trp-cage (f)	50	16/linear (1–0.5)
Trp-cage (u)	60	12/linear (1–0.5)
CLN025 (f)	15	12/linear (1–0.5)
CLN025 (u)	50	12/linear (1–0.5)
Chignolin (f)	15	12/linear (1–0.5)
Chignolin (u)	50	12/linear (1–0.5)

### Analysis

#### Structural analysis

MD trajectories were analyzed with the Python package MDTraj/1.10.3.^109^ Analysis includes the computation of dihedral angles (using the *compute_dihedrals* function), C_α_-RMSD calculation (using the *rmsd* function), and radius of gyration (using the *compute_rg* function). Free-energy landscapes were calculated using the *plot_free_energy* function implemented in the Python package PyEMMA/2.5.12.^110^

The “distance RMSD” (intramolecular distance RMSD, RMSD_dist_) was computed based on all pairwise interatomic distances in a trajectory frame and those in the reference structure. The metric is alignment-free and was calculated using only selected atoms (default: non-hydrogen atoms). For a system containing *N* selected atoms, all unique atom pairs (*i* < *j*) were considered. Pairwise distances were computed using the *compute_distances* function for both the reference structure and each trajectory frame. RMSD_dist_ was then obtained as,5
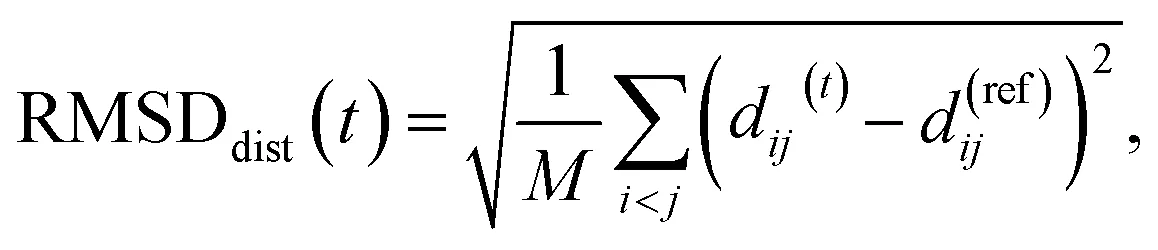
where 
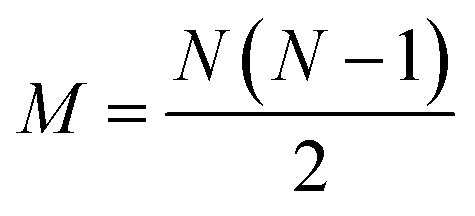
 is the total number of unique atom pairs, *d*_*ij*_^(*t*)^ is the distance between atoms *i* and *j* at frame *t*, and *d*^(ref)^_*ij*_ is the corresponding distance in the reference structure.

#### NOE violations

All NOE distances were calculated from the MD trajectories using the *r*^−6^ averaging scheme, which reflects the distance dependence of the NOE interaction. Distances between the relevant atom pairs were computed for each trajectory frame using the *compute_distances* function implemented in the Python library MDTraj/1.10.3.^109^ The effective NOE distance *r*_eff_ was then obtained according to,6
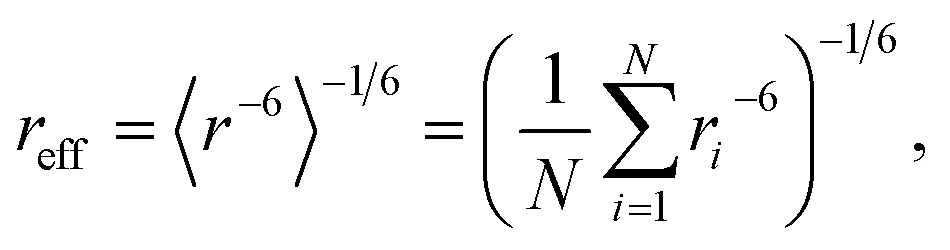
where *r*_*i*_ is the instantaneous distance between the atom pair in frame *i*, and *N* is the total number of frames considered. For each system, the analysis was carried out using the full trajectory of replica 1. For the CLN025 simulation started from the extended structure, the first 50% of the frames were excluded, as they correspond to the initial folding phase and do not represent the equilibrium ensemble.

## Author contributions

Conceptualization: R. S., I. G., and S. R.; methodology: R. S., I. G.; investigation: R. S., I. G.; visualization: R. S., I. G.; funding acquisition: S. R.; administration: S. R.; supervision: S. R.; writing—original draft: R. S., I. G., and S. R.; writing—review and editing: R. S., I. G., and S. R.

## Conflicts of interest

There are no competing interests to declare.

## Supplementary Material

SC-OLF-D6SC04555C-s001

## Data Availability

All the starting coordinates, configuration files and code for running REST2-AMP/MM simulations can be found at: https://github.com/rinikerlab/rest2_ampmm. Original data (*e.g.*, raw MD data) are available upon request to the corresponding author. Supplementary information (SI): additional methodological details; validation of AMP-BMS against QM/MM reference calculations; REST2 scaling, replica-exchange and round-trip analyses; additional Ramachandran and free-energy landscapes; convergence analyses; NOE analyses; and computational performance data. See DOI: https://doi.org/10.1039/d6sc04555c.
