# Supplementary material for: Quantum-accurate enhanced sampling and mini-protein folding through replica exchange for multiscale neural network potentials
Source: Chem Sci. 2026 Nov 9. Online ahead of print. doi: 10.1039/d6sc04555c (PMC13747000; doi:10.1039/d6sc04555c)
Supplement: Supplementary file 1 [file SC-OLF-D6SC04555C-s001.pdf]

Supplementary Information (SI) for Chemical Science.
This journal is © The Royal Society of Chemistry

SUPPORTING INFORMATION
Quantum-Accurate Enhanced Sampling and Mini-Protein Folding through Replica Exchange for Multiscale Neural Network Potentials

Riccardo Solazzo,[a,†] Igor Gordiy,[a,†] and Sereina Riniker*[a]

[a] *Department of Chemistry and Applied Biosciences, ETH Zürich, Vladimir-Prelog-Weg 2, 8093 Zürich, Switzerland. E-mail: sriniker@ethz.ch*
[†] *Authors contributed equally.*

Contents

1 Acceptance-Rate Analysis **S2**

2 Round-Trip Analysis **S2**

3 Reference QM/MM Calculations **S2**

4 Additional Figures **S3**

5 Convergence Analysis **S10**
- 5.1 Free-Energy Difference in Alanine Dipeptide . . . S10
- 5.2 CustomTABS Analysis . . . S11

6 Computational Performance **S13**

1 Acceptance-Rate Analysis

Exchange efficiency was quantified by computing both the global and pairwise acceptance rates from the REST2 log files. Each log entry reports attempted exchanges between replica pairs in the form $(i, j, \mathrm{True}/\mathrm{False})$, indicating whether the swap between replicas i and j was accepted. For each iteration block, the acceptance percentage was calculated as the ratio of accepted to attempted exchanges. The global acceptance rate was then obtained as the mean acceptance percentage over all iteration blocks. This measure reflects the overall exchange efficiency across the entire replica ladder.

In addition, pair-specific acceptance rates were computed by accumulating accepted and attempted exchanges separately for each replica pair (i, j) over the full simulation. For each pair, the acceptance rate was determined as

$$P_{\mathrm{acc}}(i,j) = \frac{N_{\mathrm{acc}}(i,j)}{N_{\mathrm{att}}(i,j)} \times 100\%, \tag{S1}$$

where $N_{\mathrm{acc}}(i,j)$ and $N_{\mathrm{att}}(i,j)$ denote the total number of accepted and attempted exchanges for that pair, respectively.

The global acceptance rate provides a measure of overall replica exchange efficiency, while the pair-resolved rates reveal potential bottlenecks or imbalances along the replica ladder. Acceptance-rate analysis for all the simulated systems can be found in Figure S2.

2 Round-Trip Analysis

The efficiency of replica mixing was quantified by evaluating full ladder round-trips in replica-index space. A round-trip is defined as a walker migrating from one end of the replica ladder to the opposite end and subsequently returning to its original endpoint, i.e., $0 \to N-1 \to 0$ or $N-1 \to 0 \to N-1$, where N denotes the total number of replicas. Exchange statistics were extracted from the REST2 log files, which record accepted and rejected swap attempts between replica pairs at each iteration. Walker trajectories were reconstructed deterministically by propagating accepted exchanges: initially, walker w occupies replica w; upon acceptance of an exchange between replicas i and j, the walkers at these replicas are swapped and their replica indices are updated accordingly. This procedure yields a discrete time series of visited replica indices across the simulation for each walker.

Round-trips in both directions were counted symmetrically. The total number of round-trips was computed individually for each walker, and summary statistics (mean, minimum, and maximum round-trips per walker) were determined. Round-trips analysis for all the simulated systems can be found in Figure S3.

3 Reference QM/MM Calculations

For all systems reported in Table S1, except those taken from Ref. [1], 100 conformers were extracted from the NPT equilibration trajectory. MM-zone particles were defined as those within 14 Å of any QM-zone atom. Reference calculations were performed in the QM/MM electrostatic embedding formalism using ORCA/6.0.1 [2–7] at the ωB97M-D4/ma-def2-TZVPP level of theory [8–16], with default settings. Mean errors for gradients, dipole moments, and quadrupole moments were computed over all Cartesian components and all conformers. Relative conformer energies were evaluated within batches of 10 conformers. This means that the reported mean absolute error for relative conformer energies was averaged over 90 structures.

Table S1: MAE of energy (kJ mol^{-1} atom^{-1}), gradients on ML-zone atoms (kJ mol^{-1} Å^{-1}), dipole moments (eÅ), and quadrupole moments (eÅ^2) across all systems. Energies are reported per atom, gradients per atom and Cartesian component, and molecular dipole and quadrupole moments per Cartesian component, all with respect to the reference DFT method (ωB97M-D4/ma-def2-TZVPP [8–16]). (f): starting from a folded conformation; (u): starting from an unfolded, fully extended, or misfolded conformation. Systems marked with an asterisk (*) correspond to data published in Ref. [1].

System	Energy	Gradient	Dipole	Quadrupole
cis-cyclo-(Pro-Ser-Leu-Asp-Val)	0.2	3.2	0.41	0.38
trans-cyclo-(Pro-Ser-Leu-Asp-Val)	0.2	3.3	0.39	0.38
Polyproline (u)	0.1	3.0	0.81	2.67
Polyproline (f)	0.0	2.6	0.42	2.61
Trp-cage*	0.1	4.5	0.74	3.41
CLN025*	0.3	4.0	0.30	0.89
Chignolin (u)	0.1	4.4	0.51	1.74
Chignolin (f)	0.2	4.4	0.73	1.10

4 Additional Figures

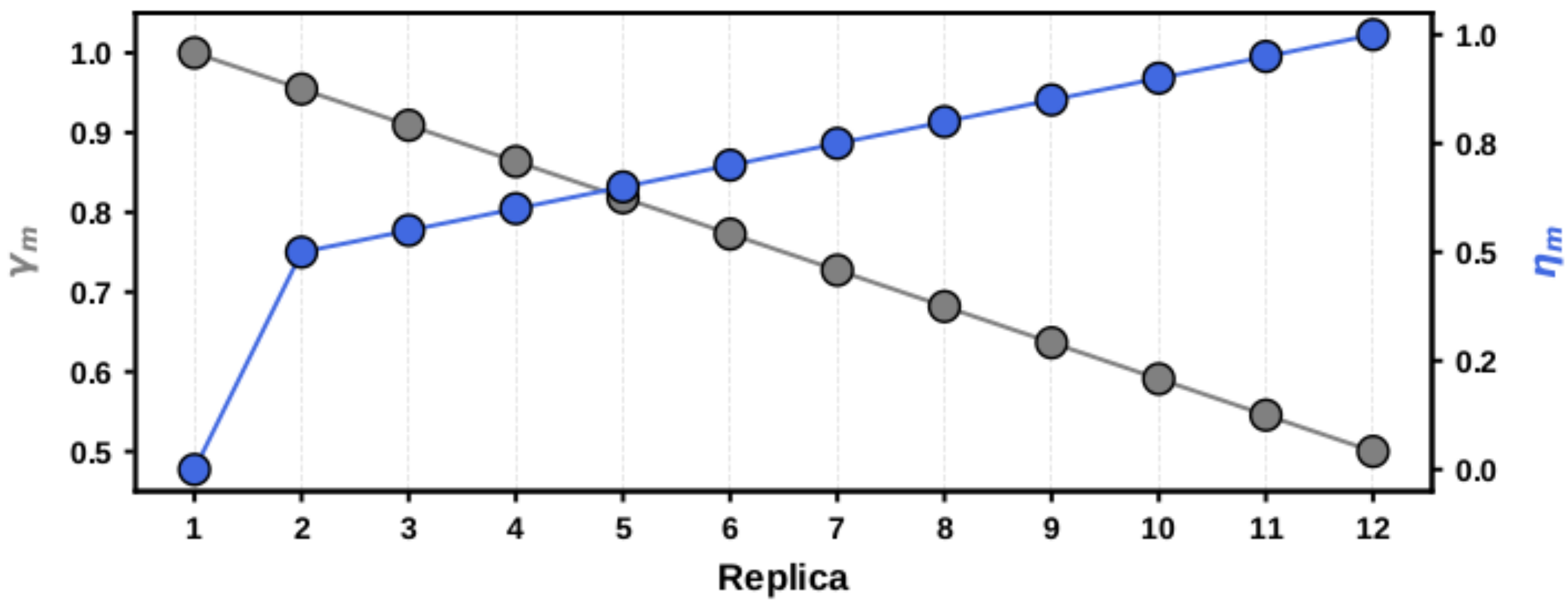

Figure S1: Replica-wise coupling between solute scaling and harmonic restraint strength in REST2. The left axis shows the solute scaling parameter γ_m (gray) applied to the intramolecular ML energy across replicas, with $\gamma_1 = 1$ corresponding to the unscaled reference replica (replica 1). The right axis reports the scaling factor for the force constant of the harmonic restraints η_m (blue). Replica 1 remains unrestrained ($\eta_1 = 0$), while replicas with stronger solute tempering (smaller γ_m) exhibit progressively increased bond and angle stabilization.

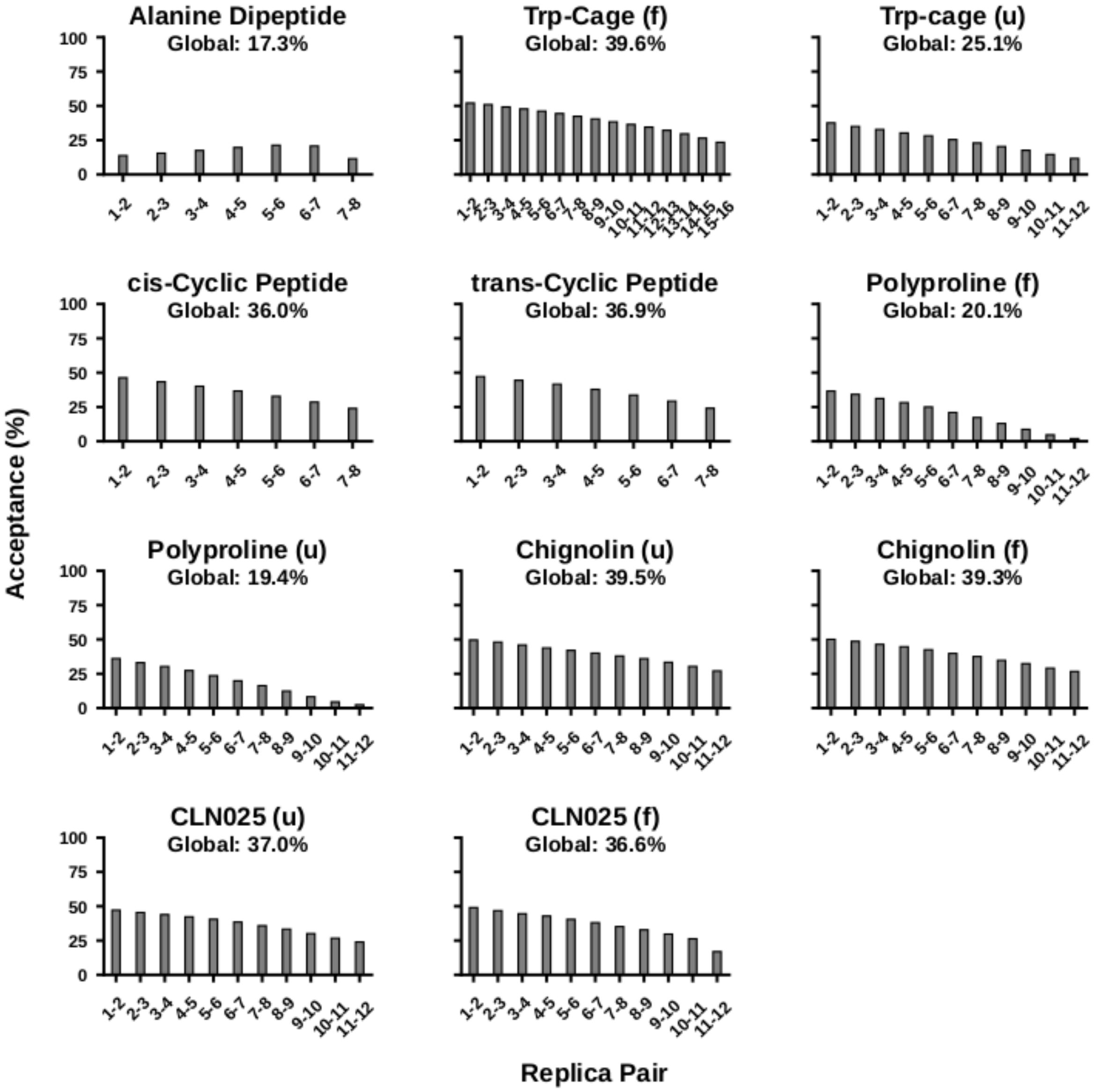

Figure S2: Replica-exchange acceptance per replica pair for all systems. Exchange-acceptance percentages between adjacent replica pairs are shown for each simulated system. Bars correspond to individual neighboring replica pairs (e.g., 1-2, 2-3, etc.), and values are sorted by replica index. The global exchange acceptance for each system, defined as the average acceptance across all replica pairs, is indicated in each panel title. Systems labeled (f) were initialized from the folded state, whereas (u) denotes simulations initiated from the unfolded state. The distributions illustrate the uniformity (or gradient) of exchange efficiency across the temperature ladder.

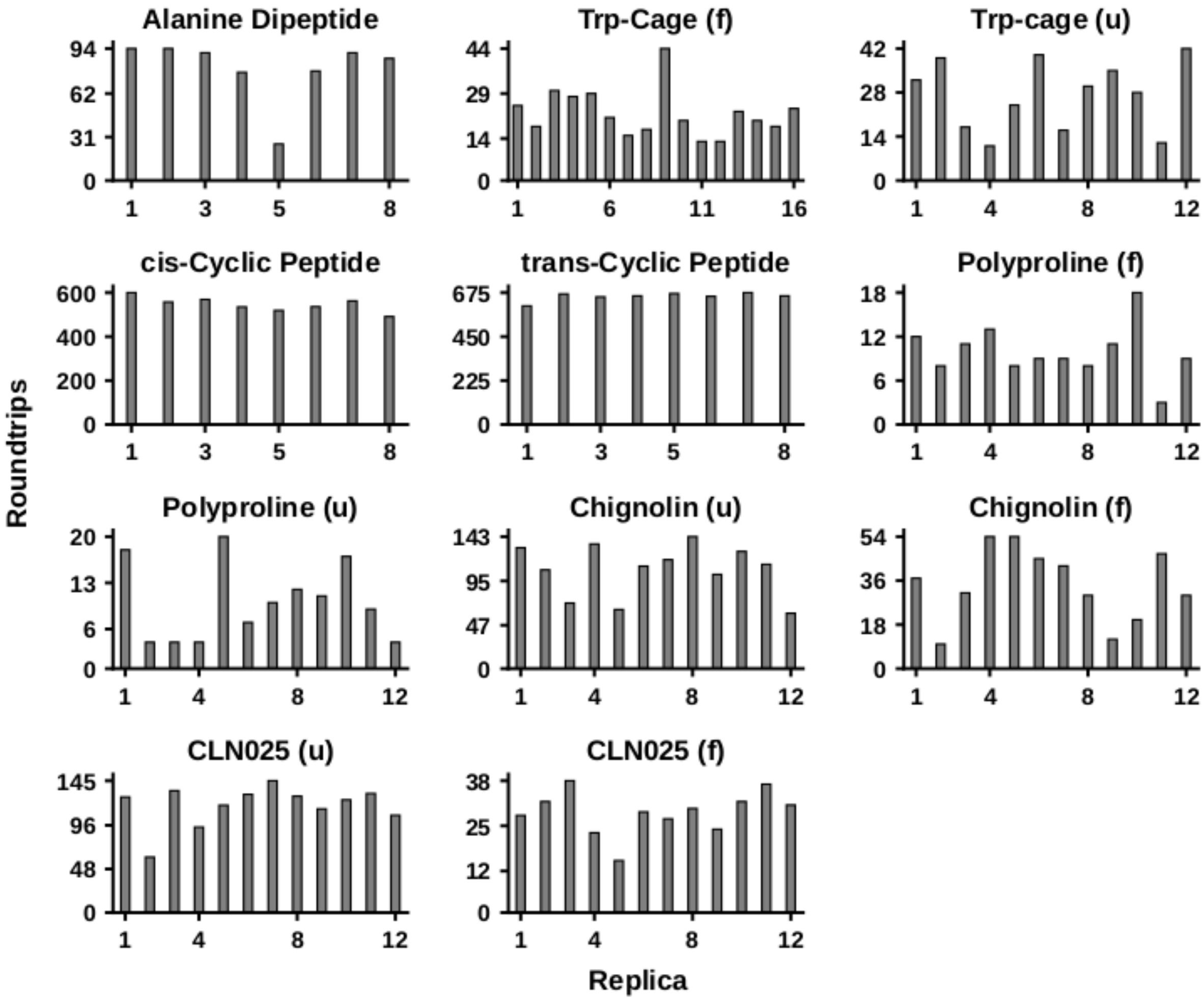

Figure S3: Round-trips per replica for all simulated systems. Number of replica round-trips observed during the simulations of Alanine dipeptide, Trp-cage mini-protein, cyclic pentapeptide, polyproline, chignolin, and CLN025. For systems simulated from both conformational basins, (f) denotes simulations initialized from the folded state and (u) from the unfolded state. Each bar corresponds to one replica in the replica-exchange ensemble. Round-trips quantify sampling efficiency and indicate how frequently replicas traverse the full temperature ladder during the production run.

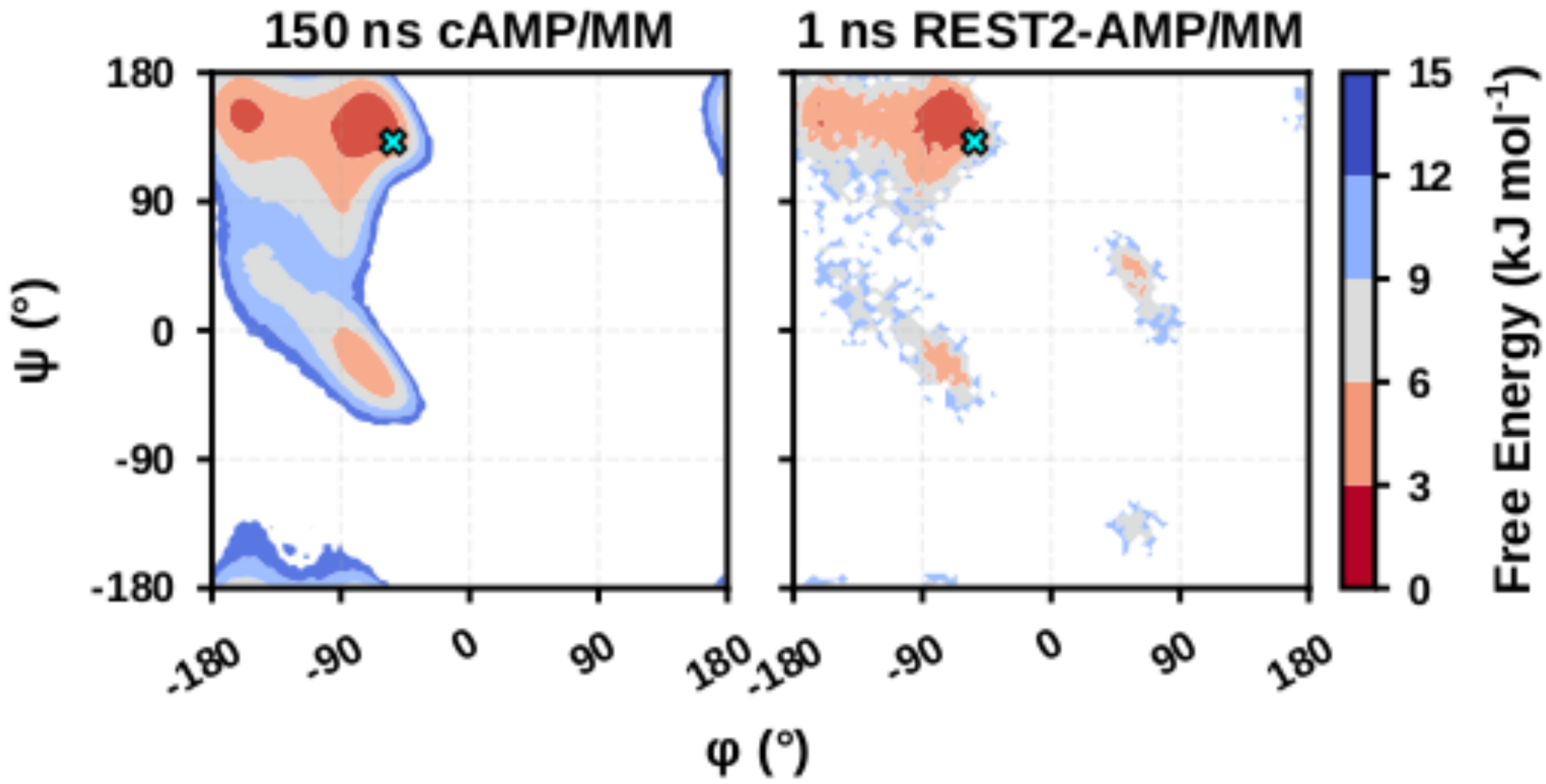

Figure S4: Conventional and REST2 simulations of alanine dipeptide. Free-energy landscapes projected onto the Ramachandran (ϕ, ψ) space for a single cAMP/MM simulation of 150 ns (left) and REST2–AMP/MM simulations (replica 1) with a total simulation time of 1 ns per replica (right). The cyan cross represents the starting conformation.

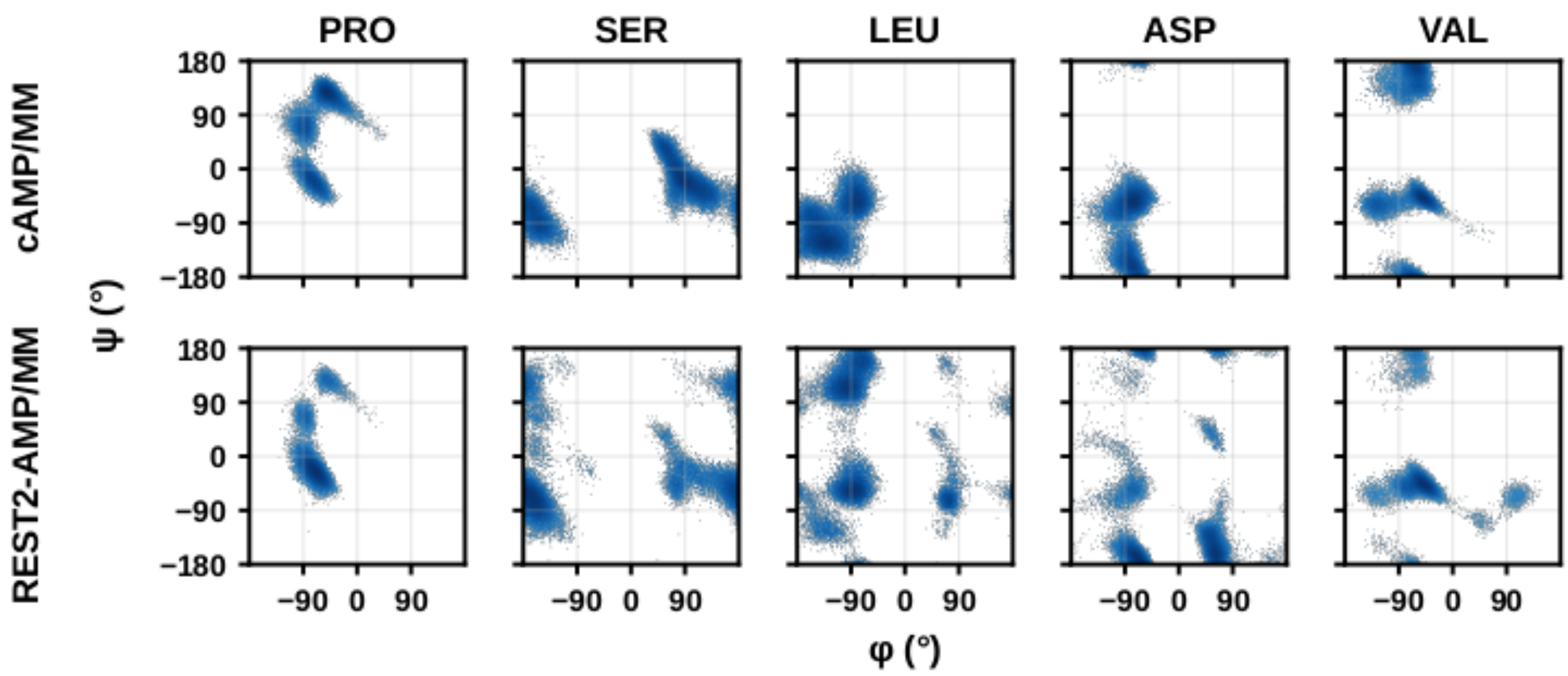

Figure S5: Backbone dihedral-angle distributions for the trans-state of the cyclic pentapeptide. Backbone dihedral-angle (ϕ, ψ) distributions for individual residues (PRO, SER, LEU, ASP, VAL) in the trans-state. The top row corresponds to simulations performed with cAMP/MM, and the bottom row to REST2-AMP/MM. Color intensity reflects the density of sampled conformations.

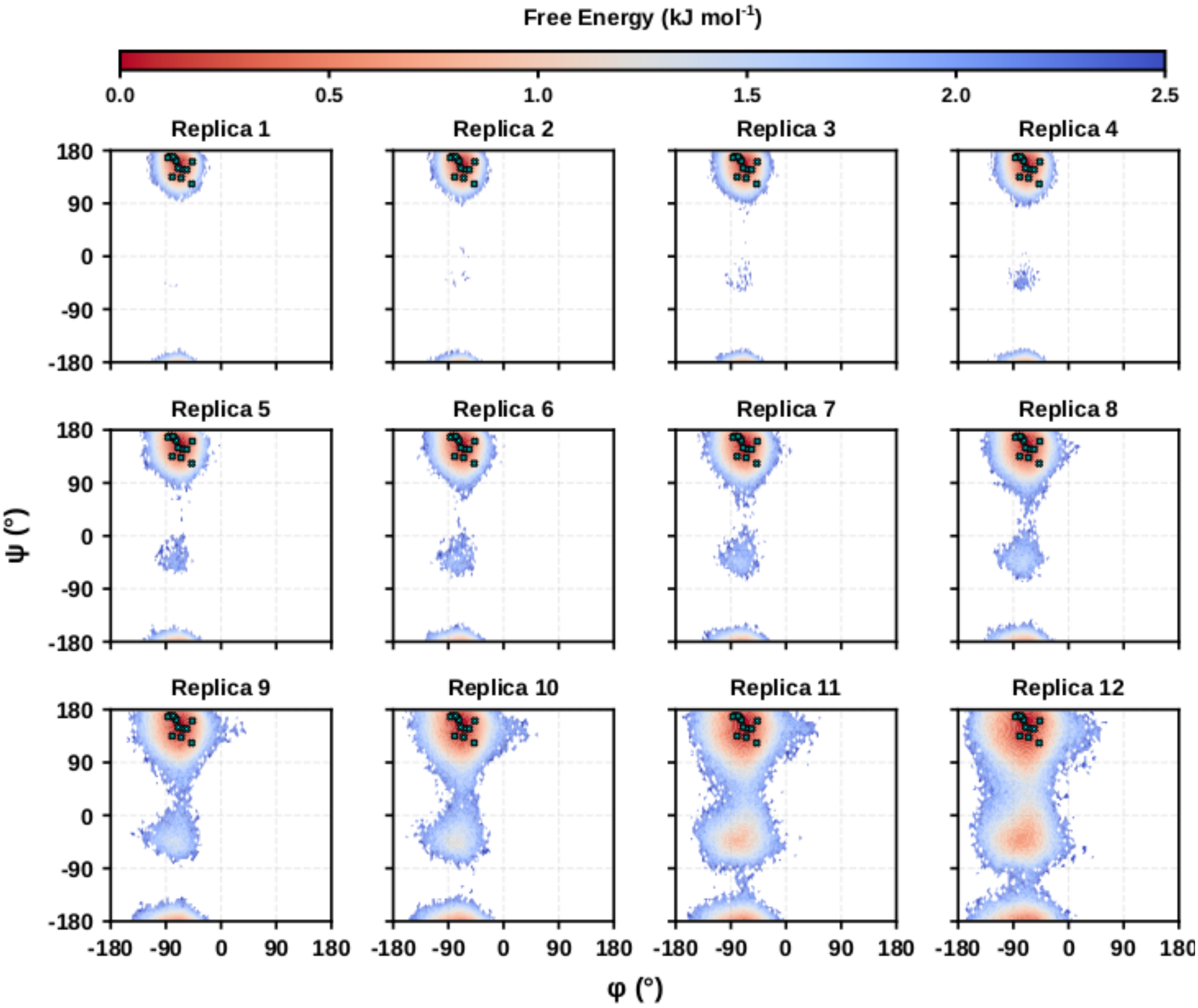

Figure S6: Backbone dihedral-angle distributions for the REST2 simulation of polyproline started in P_{II} state. Backbone dihedral-angle (ϕ, ψ) distributions shown for each replica of the REST2 simulation. The cyan crosses represent dihedral-angle values of the starting conformation.

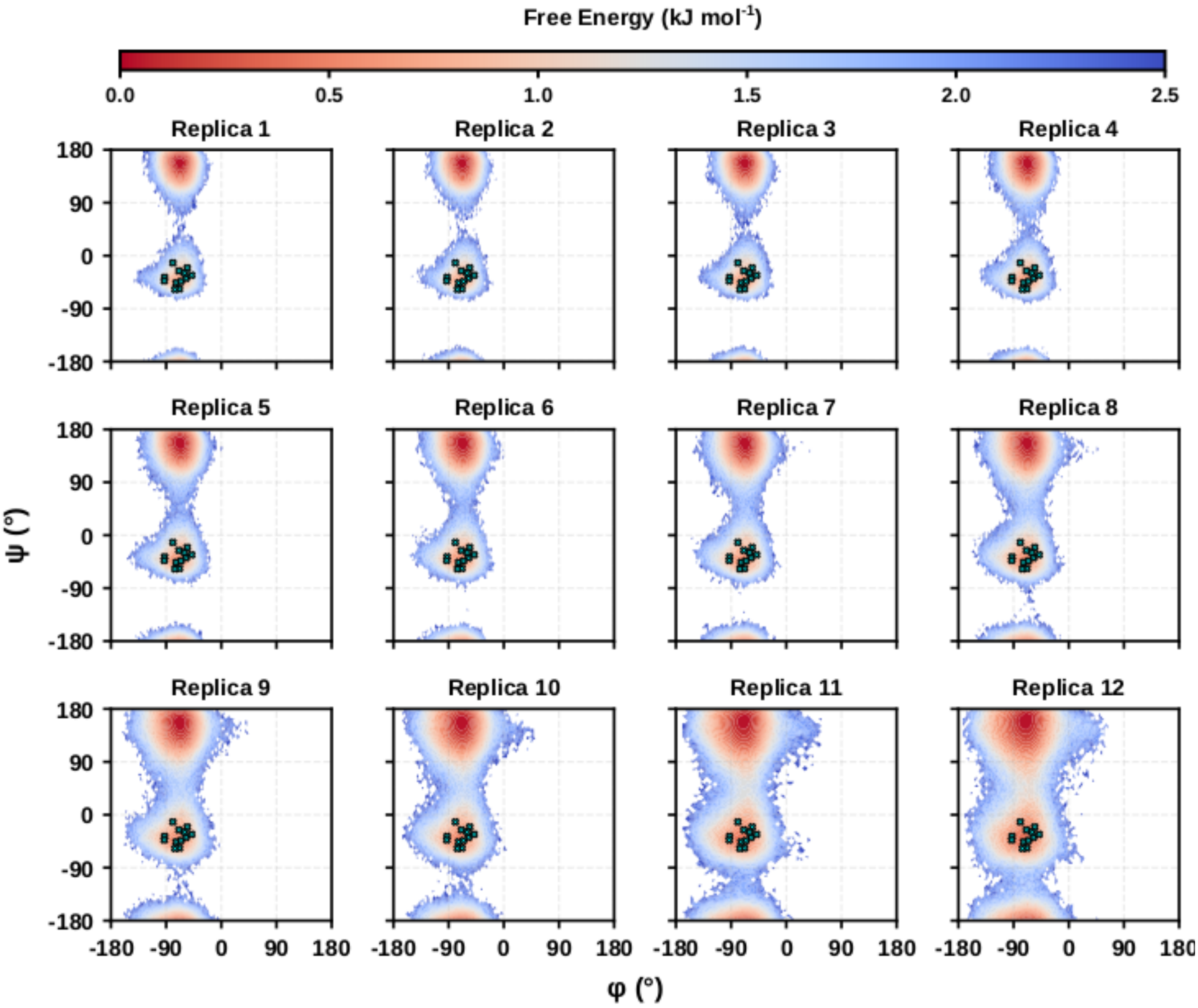

Figure S7: Backbone dihedral-angle distributions for the REST2 simulation of polyproline started in α_R state. Backbone dihedral-angle (ϕ, ψ) distributions shown for each replica of the REST2 simulation. The cyan crosses represent dihedral-angle values of the starting conformation.

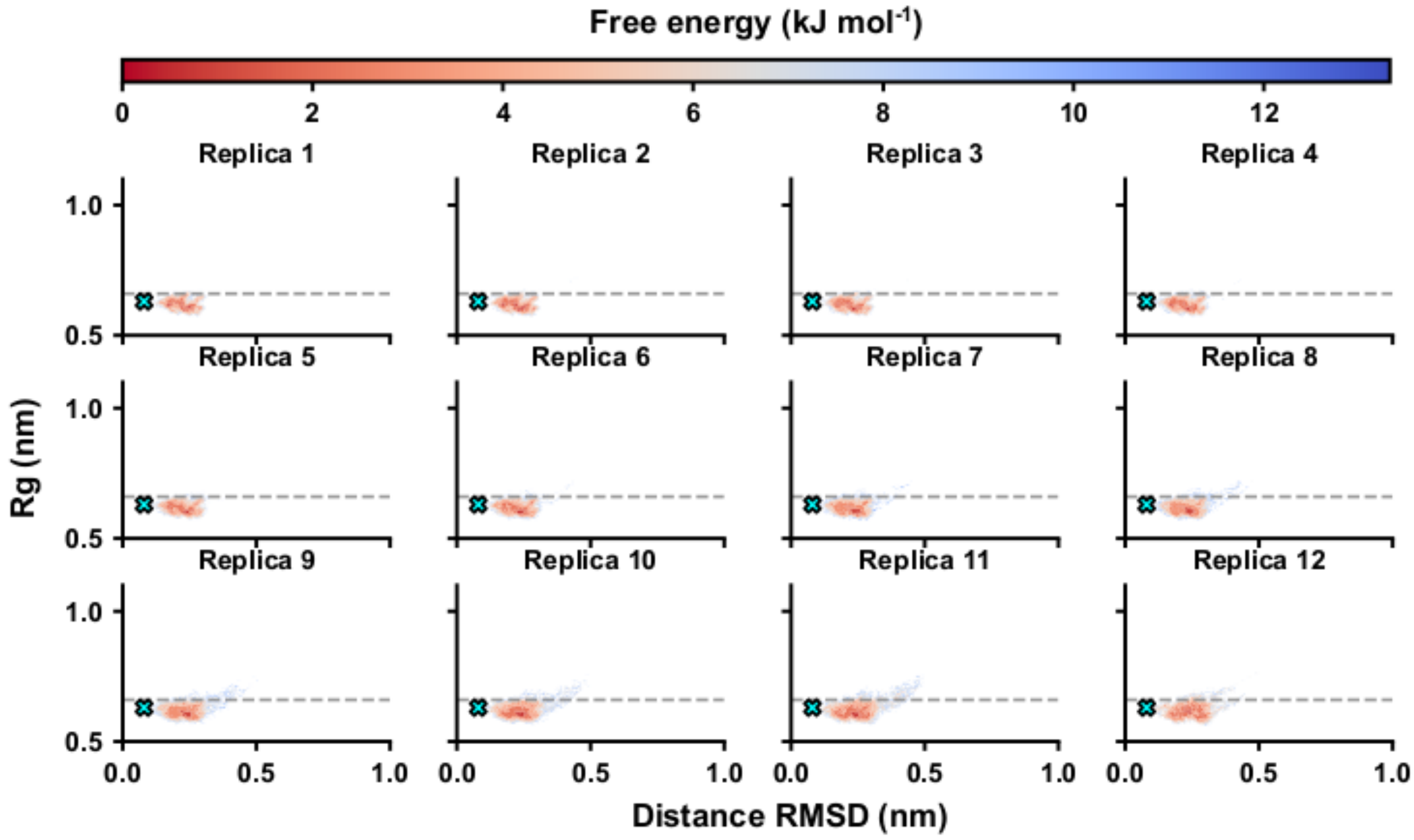

Figure S8: Free-energy landscapes of CLN025 from the REST2-AMP/MM simulation initiated from the folded structure. Free energy is shown as a function of the intramolecular distance RMSD with respect to the experimental NMR structure (PDB ID: 2RVD [17]) and the radius of gyration (R_g) for each replica. Each panel corresponds to one replica along the ladder (replicas 1-12), and the color scale reports the free energy in kJ mol^{-1}. The dashed horizontal line marks the R_g value of the compact structure. The cyan cross represents the starting conformation.

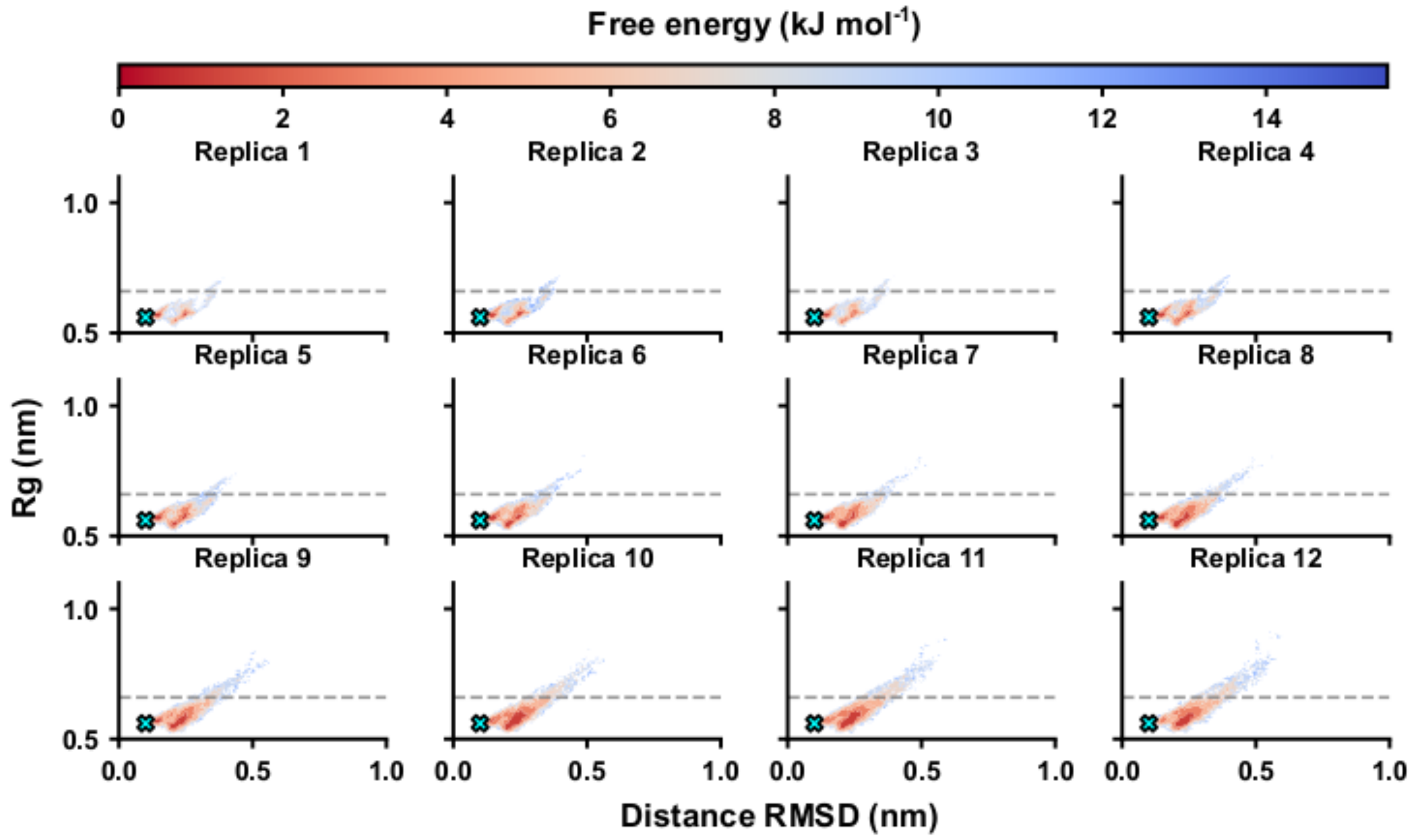

Figure S9: Free-energy landscapes of chignolin from the REST2-AMP/MM simulation initiated from the folded structure. Free energy is shown as a function of the intramolecular distance RMSD with respect to the experimental NMR structure (PDB ID: 1UAO [18]) and the radius of gyration (R_g) for each replica. Each panel corresponds to one replica along the ladder (replicas 1-12), and the color scale reports the free energy in kJ mol^{-1}. The dashed horizontal line marks the R_g value of the compact structure. The cyan cross represents the starting conformation.

5 Convergence Analysis

5.1 Free-Energy Difference in Alanine Dipeptide

Convergence was quantified using the free-energy difference between the P_{II} and α_R conformational basins of alanine dipeptide. For consistency with the polyproline analysis, the same rectangular definitions in the Ramachandran plane were adopted: the P_{II} basin was defined by $-90° \leq \phi < -40°$ and $100° \leq \psi < 180°$, whereas the α_R basin was defined by $-110° \leq \phi < -40°$ and $-90° \leq \psi < 20°$. The free-energy difference was calculated as $\Delta G_{\alpha_R - \mathrm{P_{II}}} = -RT \ln(P_{\alpha_R}/P_{\mathrm{P_{II}}})$ at 298.15 K. The complete 160 ns cAMP/MM trajectory was used as the reference and divided into ten contiguous 16 ns blocks. The uncertainty of the reference value was estimated from the standard error of the block-wise free-energy estimates using a Student-t 95% confidence interval. Cumulative free-energy differences were evaluated every 0.5 ns for cAMP/MM and the unscaled physical REST2 replica (replica 1). The convergence onset was defined retrospectively as the earliest checkpoint after which all subsequent estimates remained within the reference confidence interval, with at least three checkpoints remaining.

The resulting reference free-energy difference for the basin definitions used here was 3.35 kJ mol^{-1}, with a 95% block confidence interval of 2.59–4.12 kJ mol^{-1}. The integrated autocorrelation times were 0.018 ns and 0.072 ns for the P_{II} and α_R basin indicators, respectively, such that the 16 ns reference

blocks were approximately 222 times longer than the largest autocorrelation time. The cumulative cAMP/MM estimate reached sustained convergence after 17.5 ns, whereas the physical REST2 replica reached the same criterion after 1.5 ns, corresponding to an 11.67-fold reduction in trajectory length. Accounting for all eight REST2 replicas, the aggregate simulated time required to reach convergence was 12 ns, compared with 17.5 ns for cAMP/MM, corresponding to a 1.46-fold reduction. Using the measured production rates of 6.9 ns day^{-1} for cAMP/MM and 5.4 ns day^{-1} per REST2 replica, with all eight replicas run concurrently, the corresponding wall-clock requirements were 60.9 h and 6.7 h, respectively, representing a 9.1-fold wall-clock speedup. Benchmarking was conducted using a single NVIDIA RTX 3090 GPU for the cAMP/MM simulation and eight NVIDIA RTX 3090 GPUs for the REST2-AMP/MM simulation.

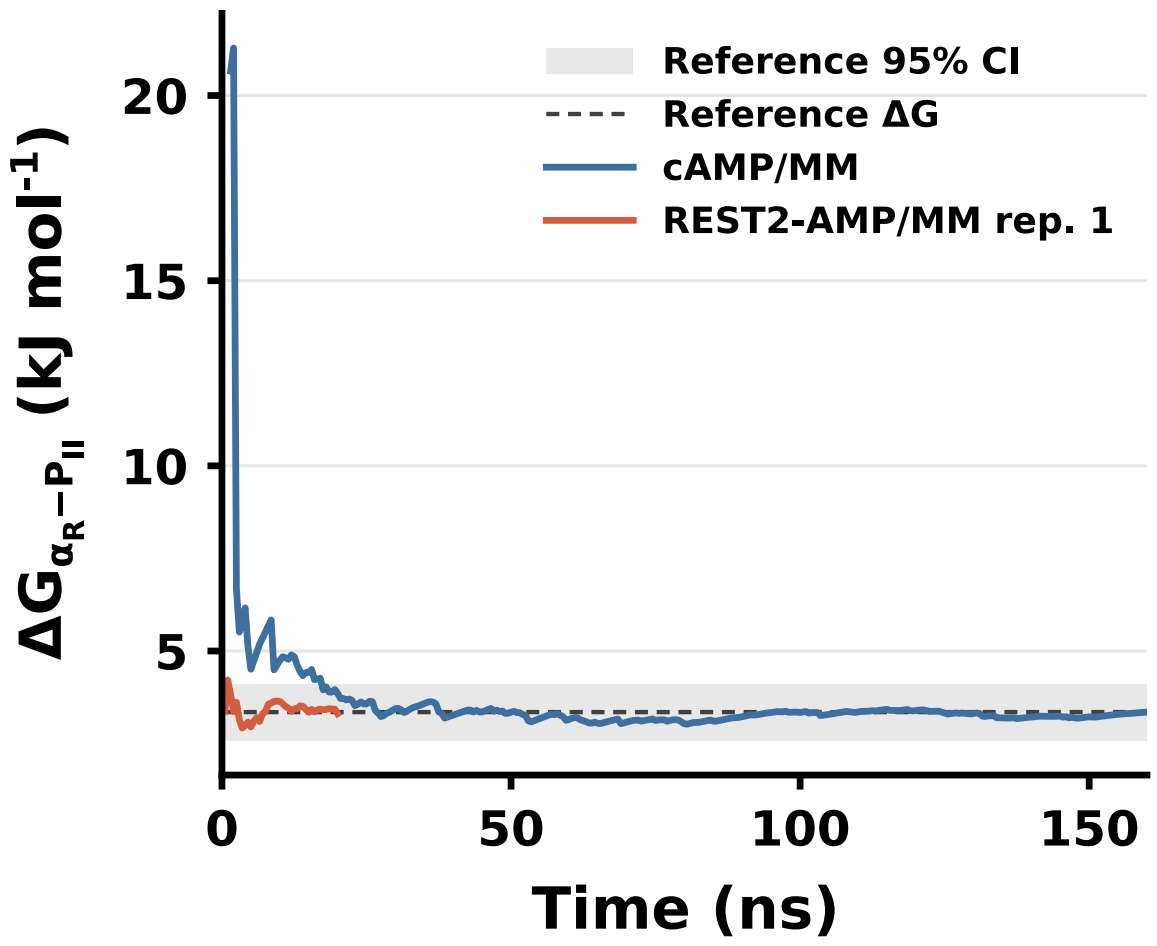

Figure S10: Convergence of the free-energy difference between the α_R and P_{II} basins of alanine dipeptide for cAMP/MM and REST2-AMP/MM. The free-energy difference, $\Delta G_{\alpha_R - \mathrm{P_{II}}} = -RT \ln(P_{\alpha_R}/P_{\mathrm{P_{II}}})$, was evaluated cumulatively as a function of trajectory time. The dashed horizontal line indicates the reference value obtained from the complete 160 ns cAMP/MM trajectory, while the shaded region represents the corresponding 95% confidence interval estimated by block analysis.

5.2 CustomTABS Analysis

To further assess the convergence of the sampled conformational ensembles, we performed the customTABS[19] analysis using all heavy atoms and the default settings. The cumulative relative populations of the ten most probable torsional states (customTABS labels) were monitored as a function of simulation time. Convergence is indicated by the stabilization of these populations, i.e., by the emergence of a plateau for the dominant torsional states. As shown in Fig. S11, the populations of the most probable states progressively stabilize over the course of the simulations, providing an additional indication of convergence of the sampled conformational ensembles.

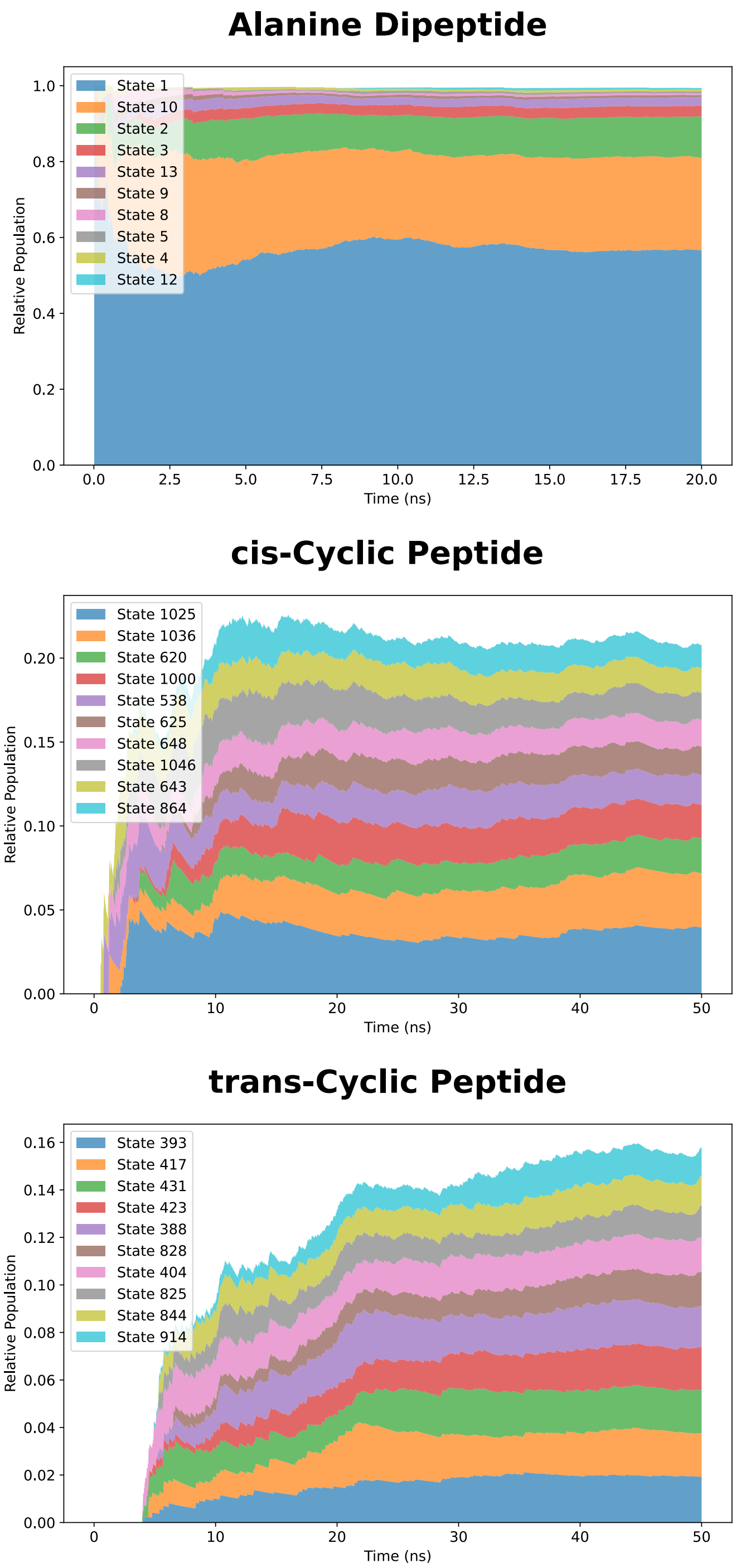

Figure S11: Cumulative relative populations of the ten most probable torsional states as defined by customTABS[19] as a function of simulation time for alanine dipeptide (top), the cis-cyclic pentapeptide (middle), and the trans-cyclic pentapeptide (bottom).

6 Computational Performance

Wall-clock performance was assessed for each simulated system by dividing the REST2-AMP/MM production trajectories into chunks of 0.5 ns and recording the real time required to complete each chunk. All simulations were performed jointly on eight NVIDIA RTX 2080Ti GPUs. Reported values (Table S2) correspond to the average wall-clock time needed to complete a full REST2-AMP/MM run of 0.5 ns for each system.

Table S2: Wall-clock time required to complete 0.5 ns of REST2-AMP/MM simulation for each system, averaged over simulation chunks of that length. All simulations were performed jointly on 8 NVIDIA RTX 2080Ti GPUs.

System	Time per 0.5 ns (h)
Alanine dipeptide	4.65
cis-cyclo-(Pro-Ser-Leu-Asp-Val)	4.32
trans-cyclo-(Pro-Ser-Leu-Asp-Val)	4.43
Polyproline (f)	5.02
Polyproline (u)	5.91
Trp-cage (f)	5.24
Trp-cage (u)	6.06
CLN025 (f)	6.42
CLN025 (u)	8.63
Chignolin (f)	5.08
Chignolin (u)	5.48

References

[1] M. Thürlemann, F. Pultar, I. Gordiy, E. Ruijsenaars and S. Riniker, *J. Am. Chem. Soc.*, 2026, **148**, 28133–28156.

[2] F. Neese, *Wiley Interdiscip. Rev.: Comput. Mol. Sci.*, 2025, **15**, e70019.

[3] F. Neese, *J. Comput. Chem.*, 2003, **24**, 1740–1747.

[4] F. Neese, F. Wennmohs, A. Hansen and U. Becker, *Chem. Phys.*, 2009, **356**, 98–109.

[5] B. Helmich-Paris, B. de Souza, F. Neese and R. Izsák, *J. Chem. Phys.*, 2021, **155**, 04109.

[6] F. Neese, *J. Comput. Chem.*, 2023, **44**, 381–396.

[7] E. F. Valeev, *Libint: A Library for the Evaluation of Molecular Integrals of Many-Body Operators over Gaussian functions*, 2022, version 2.8.0.

[8] E. R. Johnson and A. D. Becke, *J. Chem. Phys.*, 2005, **123**, 024101.

[9] O. A. Vydrov and T. Van Voorhis, *J. Chem. Phys.*, 2010, **133**, 244103.

[10] N. Mardirossian and M. Head-Gordon, *J. Chem. Phys.*, 2016, **144**, 214110.

[11] E. Caldeweyher, S. Ehlert, A. Hansen, H. Neugebauer, S. Spicher, C. Bannwarth and S. Grimme, *J. Chem. Phys.*, 2019, **150**, 154122.

[12] E. Caldeweyher, J.-M. Mewes, S. Ehlert and S. Grimme, *Phys. Chem. Chem. Phys.*, 2020, **22**, 8499–8512.

[13] A. Najibi and L. Goerigk, *J. Comp. Chem.*, 2020, **41**, 2562–2572.

[14] F. Weigend and R. Ahlrichs, *Phys. Chem. Chem. Phys.*, 2005, **7**, 3297–3305.

[15] J. Zheng, X. Xu and D. G. Truhlar, *Theor. Chem. Acc.*, 2011, **128**, 295–305.

[16] L. Wittmann, I. Gordiy, M. Friede, B. Helmich-Paris, S. Grimme, A. Hansen and M. Bursch, *Phys. Chem. Chem. Phys.*, 2024, **26**, 21379–21394.

[17] S. Honda, T. Akiba, Y. S. Kato, Y. Sawada, M. Sekijima, M. Ishimura, A. Ooishi, H. Watanabe, T. Odahara and K. Harata, *J. Am. Chem. Soc.*, 2008, **130**, 15327–15331.

[18] S. Honda, K. Yamasaki, Y. Sawada and H. Morii, *Structure*, 2004, **12**, 1507–1518.

[19] J. Braun, E. Ruijsenaars, C. Schiebroek, D. Lamei, P. Katzberger, G. A. Landrum and S. Riniker, *ChemRxiv*, 2026, DOI: 10.26434/chemrxiv.15010276.
